# Correction: Surveillance-Activated Defenses Block the ROS-Induced Mitochondrial Unfolded Protein Response

**DOI:** 10.1371/journal.pgen.1006377

**Published:** 2016-10-07

**Authors:** Eva D. Runkel, Shu Liu, Ralf Baumeister, Ekkehard Schulze

## Identification of *pifk-1* as a false screening positive

This Research Article reports that down-regulation of *pifk-1* by RNAi strongly reduces paraquat induced expression of the UPR^mt^ reporter gene *Phsp-6*::*gfp*. In their research following this publication, the authors observed that several newly generated deletion mutants of *pifk-1* did not reproduce the RNAi phenotype. Further investigation of this issue revealed that the bacterial *pifk-1* RNAi stock used in this study, in addition to the *pifk-1* RNAi plasmid, contained a minor contamination (<5%) of a second plasmid, GFP::L4440. Due to its low concentration, this contaminating plasmid was not detected in the routine Sanger sequencing. The authors suspect that the contamination was introduced into the *pifk-1* RNAi stock solution during replication of the bacterial RNAi clone, since they could not identify it in the original copy of the purchased ORFeome RNAi library. The authors confirmed by PCR analysis that the RNAi of all other 54 screening positive genes identified in their screen is free of a similar contamination.

*pifk-1* RNAi performed with a purified stock failed to prevent the induction of *Phsp-6*::*gfp* with paraquat, suggesting that *pifk-1* is not a candidate gene involved in paraquat-induced UPR^mt^. As a consequence of these new findings, the authors withdraw all results obtained with *pifk-1* RNAi in this publication and apologize for this mistake. All authors agree to the text of the correction as published here.

## Impact of the withdrawal of *pifk-1* related data from this publication

The authors report a genome-wide screen for genes required for paraquat-mediated induction of the unfolded protein response of the mitochondria (UPR^mt^). The removal of *pifk-1* from this list reduces the number of screening hits obtained after testing of 11,942 candidates from 55 to 54 hits.

The central message of the authors’ work, highlighted in the title of the publication, is that they identify a recently reported surveillance pathway of core cellular activities (Melo and Ruvkun, 2012) as a potent repressor of UPR^mt^. *pifk-1* had not been implicated in this surveillance pathway. Consequently, the withdrawal of *pifk-1*-related data does not impact the central message of this manuscript.

However, the original observations that *pifk-1* seemingly affects two unrelated organellar stress responses (UPR^mt^ and UPR^ER^) caused the authors to suggest a common membrane associated mechanism involving mitochondrial and endoplasmic membranes, a hypothesis that had not been corroborated by any additional experiments. The withdrawal of *pifk-1* involvement in UPR^mt^ eliminates the basis of this hypothesis, and, thus, diminishes the conclusions made in the publication.

The errors within the text are listed below and a revised manuscript with the *pifk-1* data removed is provided (S1 File) along with the corrected figures: Figs 6, 7, 8, 9, 10 and S5.

**Fig 6:**
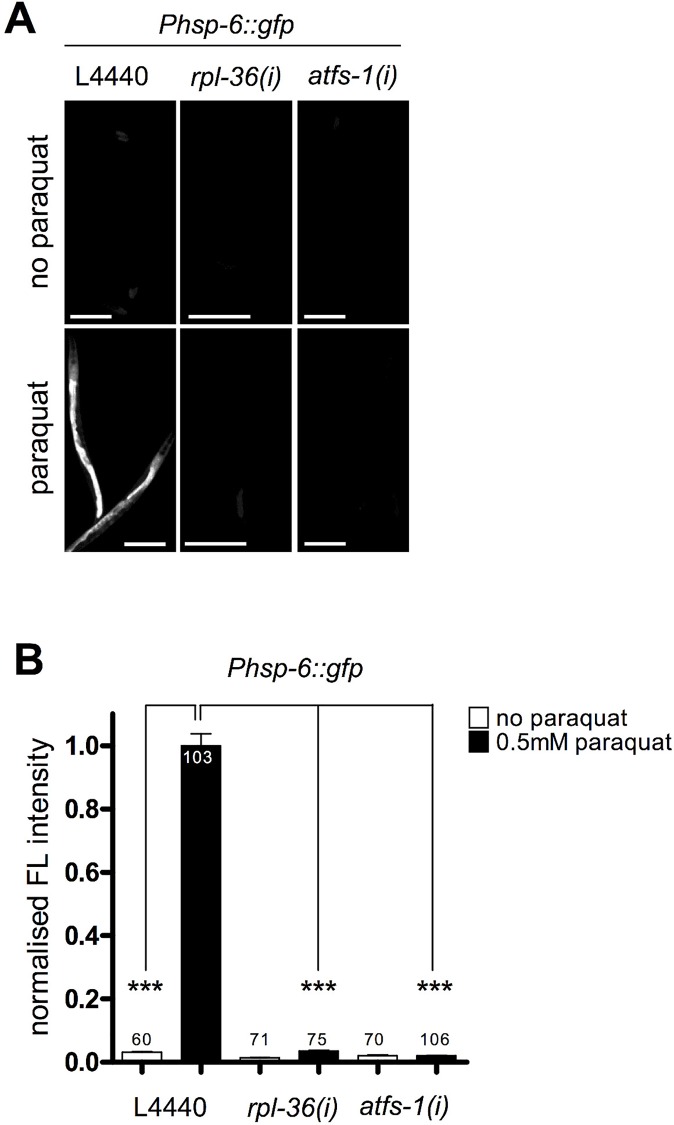
Activities of *rpl-36* and *atfs-1* are required for the *hsp-6* response to paraquat. Representative micrographs (A) and quantification of GFP fluorescence intensity (B) of two screening positives (*rpl-36*, *atfs-1*) show a block of the paraquat triggered induction of the *hsp-6* reporter (*Phsp-6*::*gfp)* after their RNAi. Worms were raised on respective RNAi plates from L1 larval stage and exposed to 0.5 mM paraquat at early L3 stage. GFP fluorescence was analyzed after two days. Columns represent pooled normalized values of three independent experiments plus standard error of the mean (SEM). Numbers in or on columns indicate the number of analyzed animals (n_total_ = 485). ***: p<0.001; Kruskal-Wallis test plus Dunn's Multiple Comparison Test; Mann Whitney test. Equal optical settings, scale bar 200 μm. *(i)*: RNAi; L4440: empty vector control.

**Fig 7:**
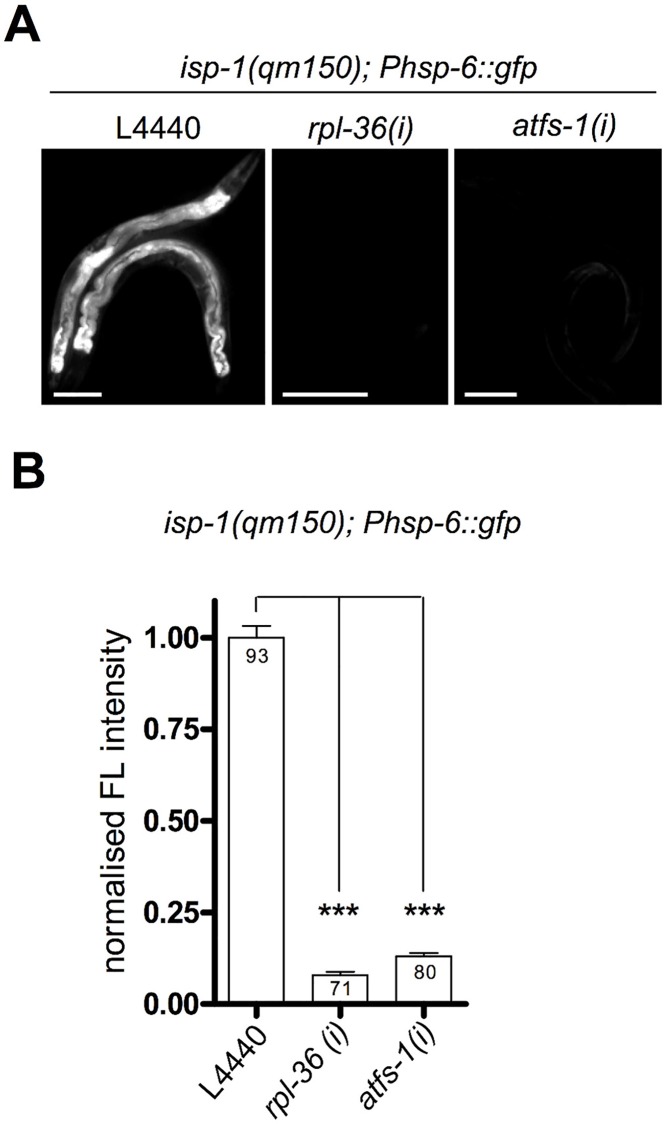
Knockdown of *rpl-36* and *atfs-1* suppresses the *isp-1(qm150)* mediated induction of the *hsp-6* reporter. The *isp-1(qm150)* mutant has increased levels of mitochondrial superoxide [17] and constitutively activated the *Phsp-6* reporter (*Phsp-6*::*gfp*). RNAi of *rpl-36* and *atfs-1* suppressed (p<0.001) the constitutive *hsp-6* reporter gene induction. Representative micrographs (A) and quantification of GFP fluorescence intensity (B). *hsp-6* reporter worms carrying the *qm150* allele were analyzed for GFP expression after one week on the respective RNAi plates. Columns represent pooled values of three independent experiments plus standard error of the mean (SEM). Numbers in columns indicate the number of analyzed animals (n_total_ = 244). ***: p<0.001; Kruskal-Wallis test plus Dunn's Multiple Comparison Test. Equal optical settings per row, scale bar 100 μm. *(i)*: RNAi; L4440: empty vector control.

**Fig 8:**
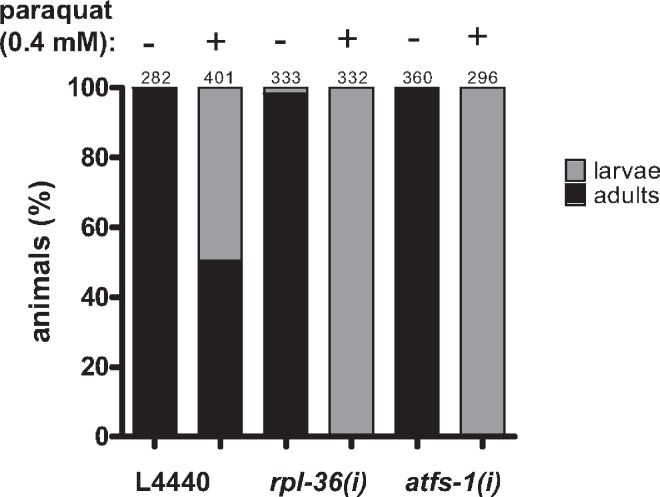
The downregulation of *rpl-36* and *atfs-1* increases paraquat sensitivity. L1 staged N2 worms were placed on the respective RNAi plates containing 0.4 mM or no paraquat, development was analyzed five days later. Downregulation of both genes enhanced sensitivity towards paraquat, indicated by delayed development. Columns represent pooled values of three independent experiments in percent. Numbers on columns indicate the number of animals analyzed (n_total_ = 2004). *(i)*: RNAi; L4440: empty vector control.

**Fig 9:**
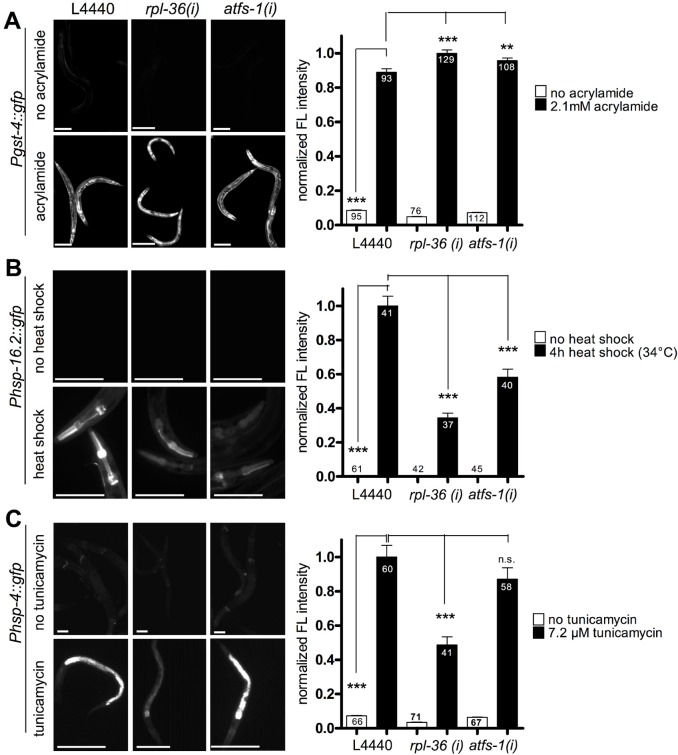
The knockdown of *rpl-36* and *atfs-1* does not prevent non-mitochondrial stress responses. Worms were grown from L1 larval stage on the respective RNAi plates before being exposed to the respective stress and analyzed four days after L1. A. A reporter strain for the SKN-1 dependent phase II response *(Pgst-4*::*gfp)* was exposed to 2.1 mM acrylamide starting at early L3 stage. RNAi of *rpl-36* and *atfs-1* did not prevent reporter gene induction as compared to vector control. Columns represent pooled normalized values of four independent experiments plus standard error of the mean (SEM). Numbers in columns indicate the number of analyzed animals (n_total_ = 613). ***: p<0.001; Kruskal-Wallis test plus Dunn's Multiple Comparison Test; Mann Whitney test (comparison of vector with and without acrylamide). Equal optical settings, scale bar 200 μm. B. Cytosolic UPR (heat shock) reporter worms *(Phsp-16*.*2*::*gfp)* were exposed to 34°C for 4h at L4. The knockdown of *rpl-36* and *atfs-1* significantly decreased heat stress induced reporter expression (p<0.001). Columns represent pooled normalized values of two independent experiments plus standard error of the mean (SEM). Numbers in or on columns indicate the number of analyzed animals (n_total_ = 266). ***: p<0.001; Kruskal-Wallis test plus Dunn's Multiple Comparison Test; Mann Whitney test (comparison of vector with and without heat shock). Equal optical settings, scale bar 100 μm. C. The UPR^ER^ reporter strain (*Phsp-4*::*gfp*) was raised from L1 stage RNAi plates (with 7.2 μg/ml tunicamycin). UPR^ER^ induction was not blocked by any RNAi tested here, but *rpl-36* (RNAi) strongly impaired its induction (p<0.001). Columns represent pooled normalized values of four independent experiments plus standard error of the mean (SEM). Numbers in or on columns indicate the number of analyzed animals (n_total_ = 363). ***: p<0.001; Kruskal-Wallis test plus Dunn's Multiple Comparison Test; Mann Whitney test (comparison of vector with and without tunicamycin). Equal optical settings, scale bar 100 μm. *(i)*: RNAi; L4440: empty vector control.

**Fig 10:**
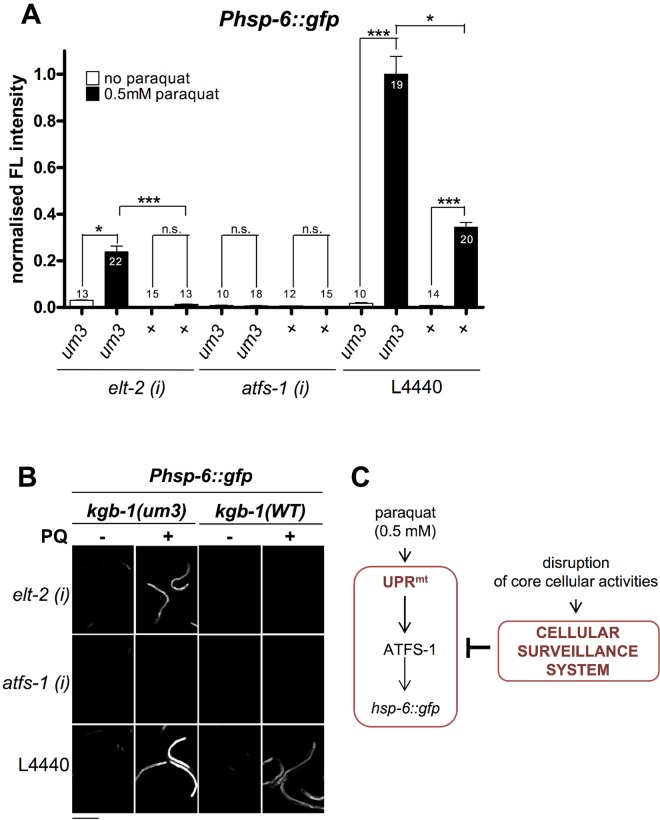
cSADDs inhibits paraquat mediated signaling to *hsp-6* through KGB-1. A-B. In *kgb-1(um3)* mutant animals, which are cSADDs deficient, paraquat induced *hsp-6* induction is not blocked by *elt-2* RNAi. Thus, ROS induced UPR^mt^ is activated in the absence of functional cSADDs. In contrast, *kgb-1(um3)* does not prevent inhibition of *hsp-6* induction by *atfs-1* knockdown, suggesting that it functions downstream of *kgb-1* and the cSADDs. Columns represent normalized values plus standard error of the mean (SEM). Numbers in or on columns indicate the number of analyzed animals (n_total_ = 181). ***: p<0.001, *: p<0.05; Kruskal-Wallis test plus Dunn's Multiple Comparison Test (A). Equal optical settings, scale bar 400 μm. *(i)*: RNAi; L4440: empty vector control; +: wild type allele (B). C. Model: Genes activating the cSADDs (cellular surveillance system) inhibit the paraquat-triggered induction of the UPR^mt^.

## Abstract

“In addition, we identified PIFK-1, the orthologue of the Drosophila PI 4-kinase four wheel drive (FWD), and found that it is the only known factor so far that is essential for the unfolded protein responses of both mitochondria and endoplasmic reticulum. This suggests that both UPRs may share a common membrane associated mechanism.”

The authors withdraw the hypothesis of a common membrane associated mechanism for both UPRs.

## Introduction

*“pifk-1* encodes a novel PI 4-kinase, downregulation of which blocks the UPR^mt^ independently of the surveillance pathway and may, therefore act downstream of it”

*The authors withdraw the identification of* pifk-1 *as a regulator of* UPR^mt:^.

## Results

*pifk-1* RNAi prevents *hsp-6*::*gfp* induction by paraquat (Table 1 in S1 File, Fig 6).

*pifk-1* RNAi prevents *hsp-60*::*gfp* induction in the *zc32* mutant (S5 Fig).

*pifk-1* RNAi prevents *hsp-6*::*gfp* induction in the *isp-1(qm150*) mutant (Fig 7).

*pifk-1* RNAi increases paraquat sensitivity (Fig 8).

*pifk-1* RNAi reduces reporter gene inductions by acrylamide and tunicamycin (*gst-4*::*gfp*, *gst-4*::*gfp*) and also the basal level of *gst-4*::*gfp* expression (Fig 9).

Of all screening positive clones only *Pifk-1* RNAi is required for the UPR^ER^ (Table 1 in S1 File).

*pifk-1* does not trigger the food aversion response (Table 2 in S1 File).

*pifk-1* RNAi both in the *kgb-1(+)* control strain and in the *kgb-1(um3)* mutant, blocks the paraquat-triggered *hsp-6*::*gfp* induction (Fig 10).

*pifk-1* has proposed signaling functions in the UPR^mt^ and in UPR^ER^.

*The authors withdraw the identification of* pifk-1 *as a regulator of UPR*^*mt*:^ *and UPR*^*ER*^. *Because* pifk-1 *RNAi did not trigger the food aversion response it was not considered as being part of this pathway*. *With respect to this*, pifk-1, *in addition to* atfs-1, *was tested as a control for the dependence on the cSADDs regulator* kgb-1. *The deleted* pifk-1 *data (**Fig* 10*) show that a non-cSADDs inducing RNAi*, *which inhibits* hsp-6::gfp *induction*, *is not dependent on* kgb-1. *Although this argument is formally still valid*, *the authors now solely refer to the evidence obtained with the confirmed UPR*^*mt*^ *regulator ATFS-1*.

## Discussion

Paragraph: PIFK-1 is a proposed new factor in UPR^mt^ and UPR^ER^ stress signaling.

*The authors withdraw the identification of* pifk-1 *as a regulator of UPR*^*mt*:^ *and UPR*^*ER*^.

## Supporting Information

S5 Fig The effects of RNAi of *rpl-36* and *atfs-1* on *zc32* mediated activation of *Phsp-60*::*gfp*. Representative micrographs (A) and quantification of GFP fluorescence intensity (B). The UPR^mt^ reporter strain (*zc32; Phsp-60*::*gfp*) induces the UPR^mt^ upon shift to the restrictive temperature (25°C). Being raised on the respective RNAi plates from L1, worms were shifted from the permissive temperature (15°C) to 25°C as soon as the animals grown on control RNAi plates (L4440) had developed to L4/young adults. GFP fluorescence was analyzed after two days. As expected, knockdown of *atfs-1* reduced GFP expression. This indicates the requirement of *atfs-1* for the UPR^mt^ (p<0.001). The induction of the UPR^mt^ was not reduced but rather enhanced by *rpl-36* RNAi (p<0.001). Columns represent pooled normalized values of four independent experiments plus standard error of the mean (SEM). Numbers in or on columns indicate the number of analyzed animals (n_total_ = 520). ***: p<0.001; Kruskal-Wallis test plus Dunn's Multiple Comparison Test; Mann Whitney test (comparison of vector at 15°C and 25°C). Equal optical settings, scale bar 200 μm. *(i)*: RNAi; L4440: empty vector control.
(EPS)

S1 File Updated manuscript with references to *pifk-1* data removed (DOCX)
