# Supplementary figures and images for: Surveillance-Activated Defenses Block the ROS–Induced Mitochondrial Unfolded Protein Response
Source: PLoS Genet. 2013 Mar 14;9(3):e1003346. doi: 10.1371/journal.pgen.1003346 (PMC3597513; doi:10.1371/journal.pgen.1003346)

Figure S1

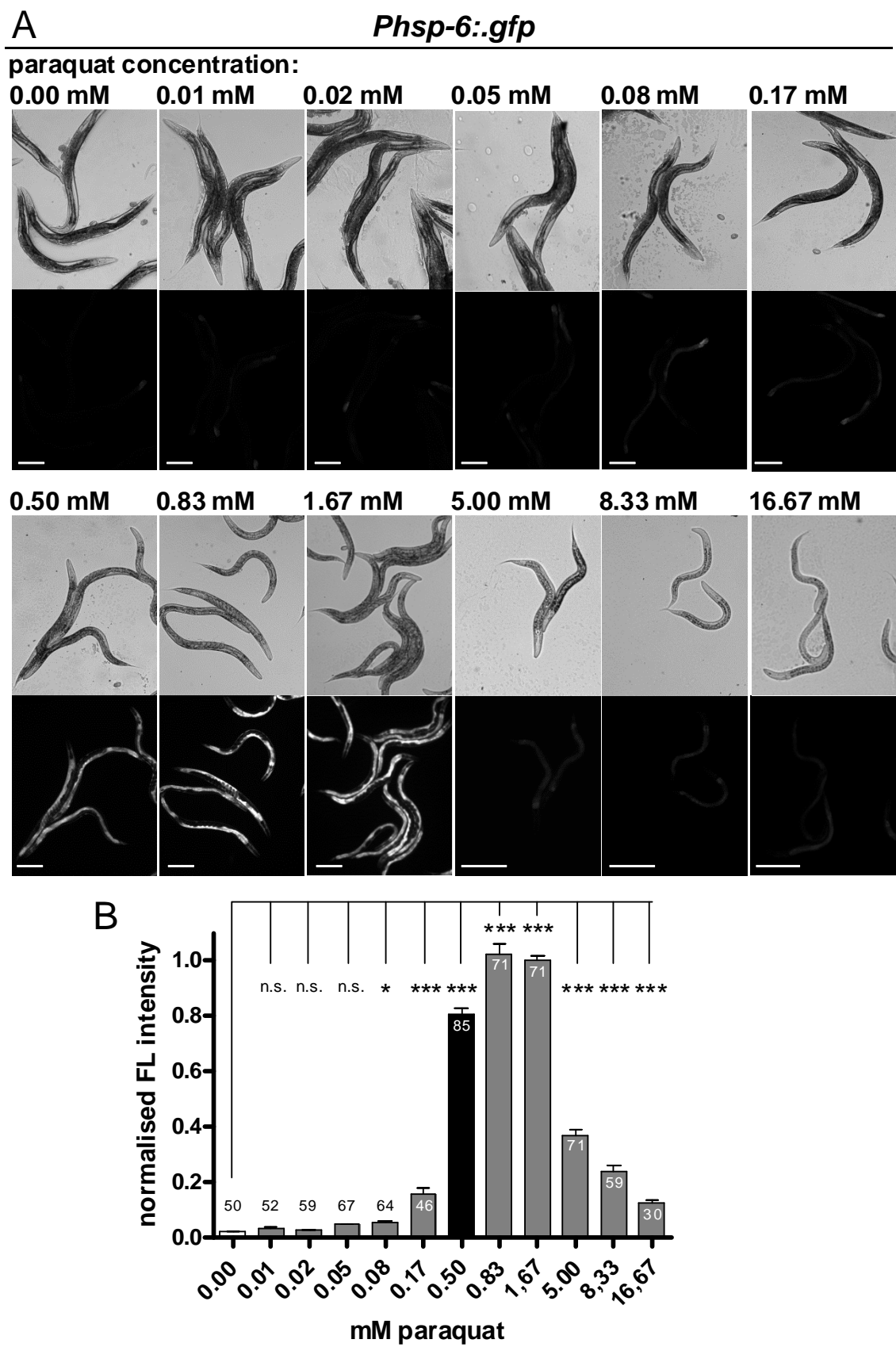

Supplement: Figure S1 — Dose-response curve of paraquat and Phsp-6::gfp. Phsp-6::gfp reporter worms were exposed to different concentrations of paraquat (0–50 mM) for two days starting from early L3. 50 µM was lethal. GFP fluorescence intensity was analyzed with compound microscopy. A. Representative micrographs. B. Corresponding quantification. Columns represent pooled values of three independent experiments plus standard error of the mean (SEM). Numbers in columns indicate the number of analyzed animals (ntotal = 725). ***: p<0.001; Kruskal-Wallis test plus Dunn's Multiple Comparison Test. (PDF) [file pgen.1003346.s001.pdf]

Figure S2

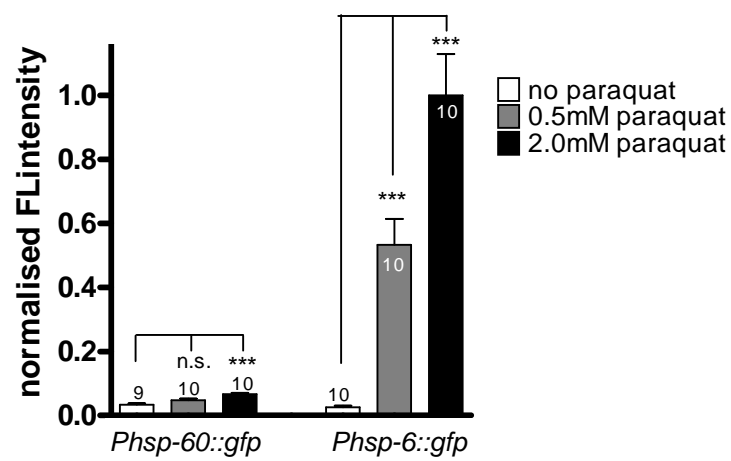

Supplement: Figure S2 — Phsp-6::gfp responds more sensitively to paraquat than Phsp-60::gfp. Quantification of GFP fluorescence intensity in the hsp-6 reporter strain (Phsp-6::gfp) and the hsp-60 reporter strain (Phsp-60::gfp) after two days of exposure to 0.5 mM and 2.0 mM paraquat, respectively. Exposure started at the early L3 stage. 0.5 mM Paraquat significantly increases (p<0.0001) hsp-6 reporter expression, but not Phsp-60::gfp, 2.0 mM paraquat induces both reporters. Columns represent mean plus standard error of the mean (SEM). Numbers in or on columns indicate the number of analyzed animals (hsp-6: ntotal = 30; hsp-6: ntotal = 29). ***: p<0.001; Kruskal-Wallis test plus Dunn's Multiple Comparison Test. (PDF) [file pgen.1003346.s002.pdf]

Figure S3

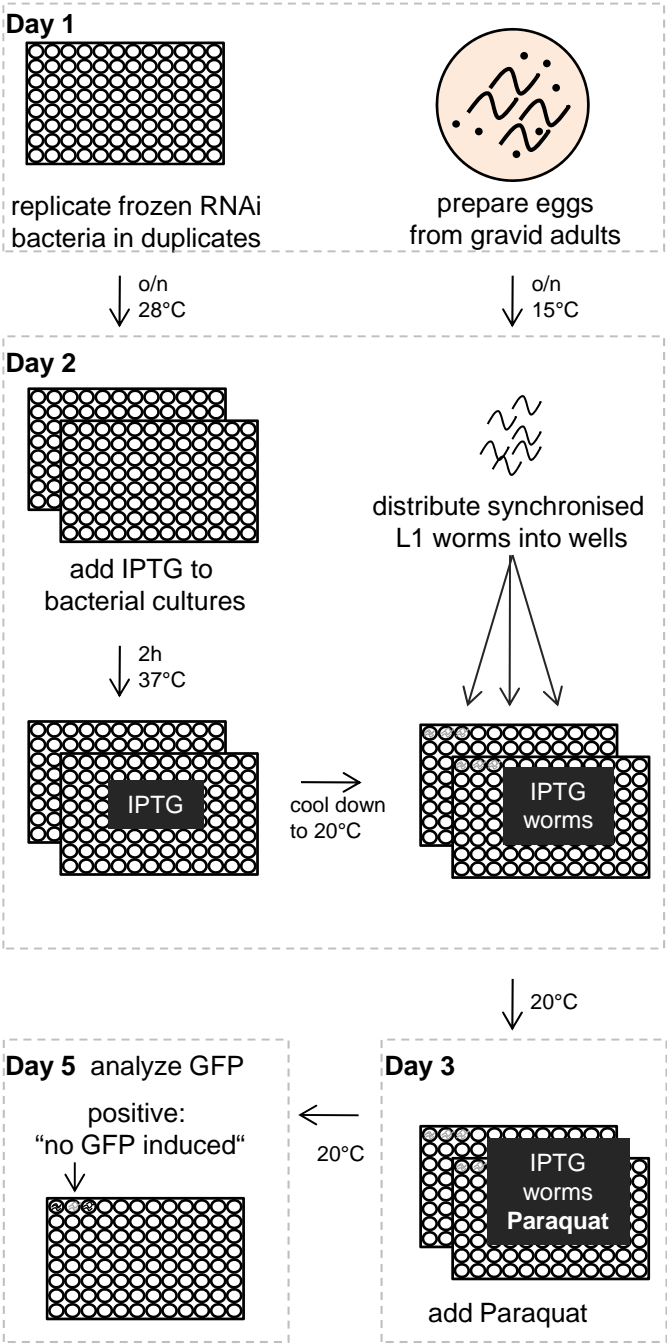

Supplement: Figure S3 — Design of the genome-scaled RNAi screen. Microtiter plates including RNAi bacterial strains were grown overnight in duplicates. Each well contained RNAi bacteria specific for one C. elegans gene. The same day, eggs were prepared by bleaching gravid adults and allowed to further develop overnight in supplemented M9. The next day (Day 2), bacterial cultures were induced with IPTG. Subsequently, synchronized L1 larvae were added to the bacterial cultures and maintained at 20°C. At Day 3, paraquat was added. After two days (Day 5), plates were screened for worms that failed to increase GFP expression with a stereo fluorescence microscope. (PDF) [file pgen.1003346.s003.pdf]

**Figure S4**

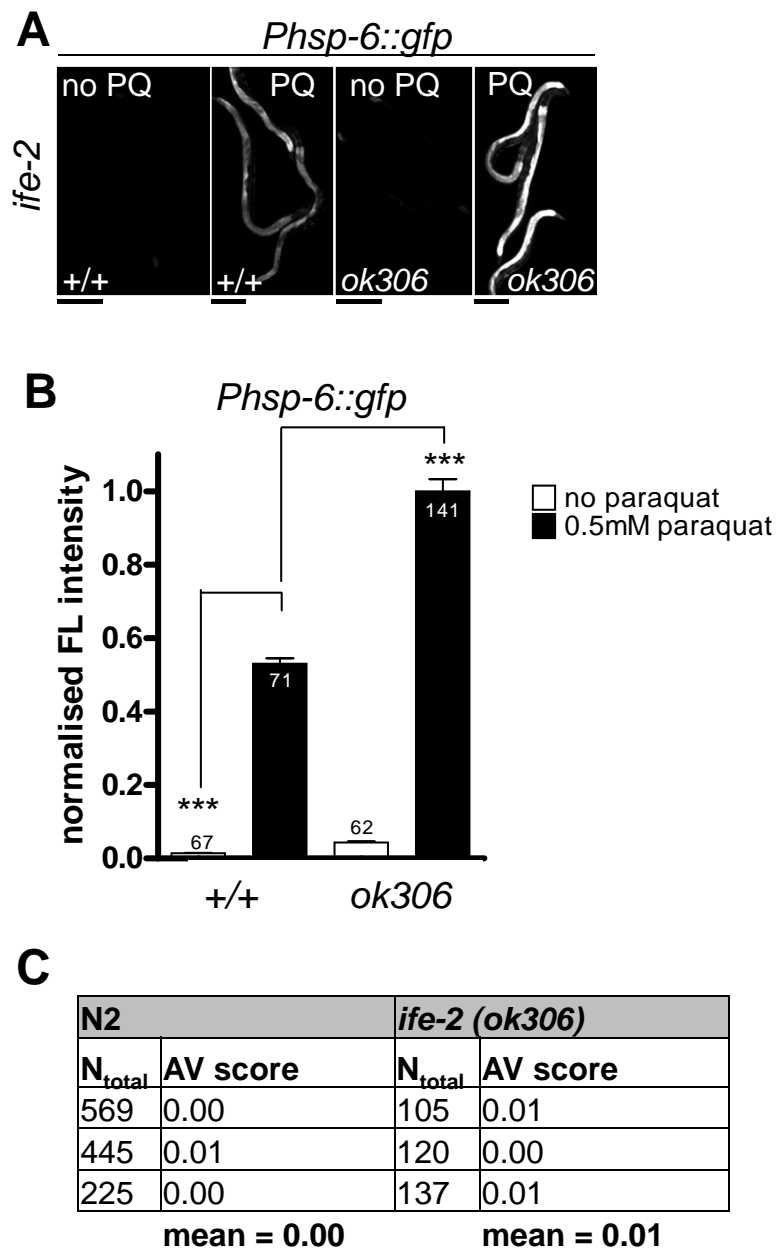

Supplement: Figure S4 — Knockdown of translation associated genes abolishes hsp-6 induction without blocking translation. The largest group of screening positives corresponds to genes encoding ribosomal proteins or other factors implicated in protein translation. In the most trivial scenario RNAi against translation associated genes reduces translation and thus prevents GFP expression from the hsp-6 reporter (A). This idea is contradicted by experiments in which the induction of other GFP reporters (hsp-16.2::gfp, gst-4::gfp, hsp-4::gfp) is still possible, when translation associated genes were knocked down (Figure 9). rpl-36 RNAi even slightly hyper-activated acrylamide induced expression of gst-4::gfp (Figure 9A). It is therefore unlikely that general translation is largely hampered by downregulation of ribosomal genes in our experiments. To further affirm this conclusion we took advantage of the ife-2(ok306) deletion [68] mutant, in which somatic translation is reduced. This mutant should mimic the effect of translation associated RNAis if the observed inhibitory effect is mitigated through a reduction of protein translation. Paraquat induced hsp-6 expression was not reduced, but rather increased in ok306 mutant animals, indicating that a moderate inhibition of translation is not sufficient to prevent hsp-6 induction by paraquat. The hyper-induction of hsp-6::gfp could be explained by an aggravation of stress caused by reduced translation of chaperones. Taken together, we consider it unlikely that RNAi against ribosomal genes substantially reduces translation and thereby prevents hsp-6 reporter expression. The results rather indicate a selective inhibitory response to hsp-6::gfp induction. A. Representative micrographs of Phsp-6 reporter (Phsp-6::gfp) worms carrying the ife-2(ok306) allele induced with paraquat. ok306 causes a moderate reduction of general protein translation without causing an imbalance of ribosomal proteins [68], [69]. The hsp-6 induction was not reduced in an ok306 a [file pgen.1003346.s004.pdf]

Figure S5

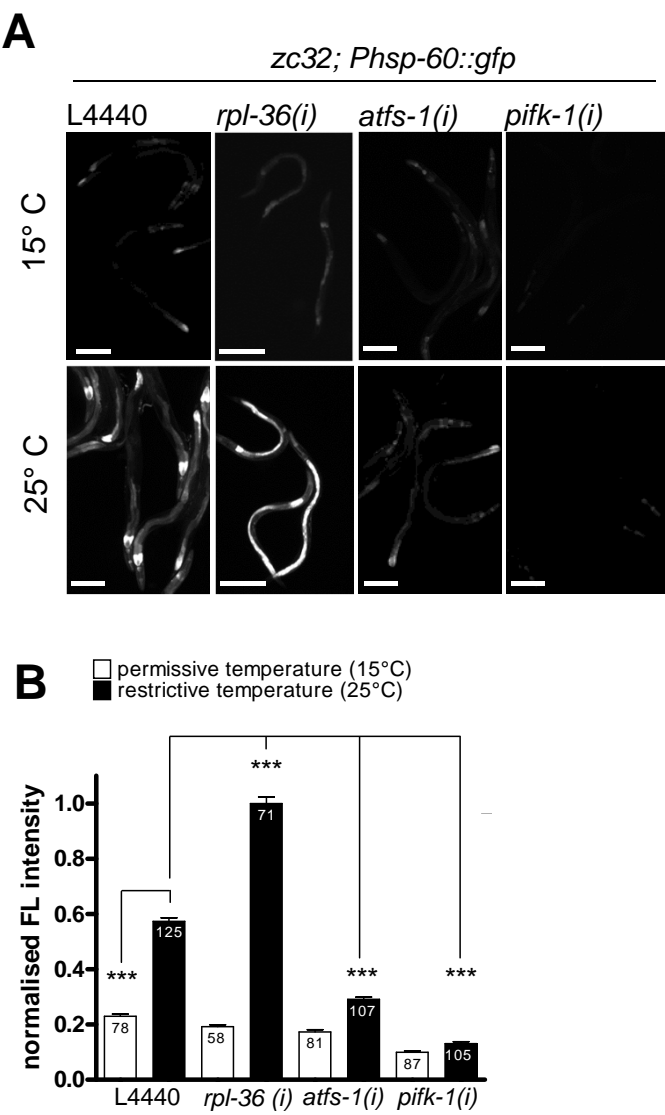

Supplement: Figure S5 — The effects of RNAi of rpl-36, atfs-1 and pifk-1 on zc32 mediated activation of Phsp-60::gfp. Representative micrographs (A) and quantification of GFP fluorescence intensity (B). The UPRmt reporter strain (zc32; Phsp-60::gfp) induces the UPRmt upon shift to the restrictive temperature (25°C). Being raised on the respective RNAi plates from L1, worms were shifted from the permissive temperature (15°C) to 25°C as soon as the animals grown on control RNAi plates (L4440) had developed to L4/young adults. GFP fluorescence was analyzed after two days. While the induction of the UPRmt was enhanced by rpl-36 RNAi (p<0.001), a complete block of the UPRmt was observed by RNA interfering with the PI 4-kinase gene pifk-1. This indicates the requirement of atfs-1 for the UPRmt (p<0.001). Columns represent pooled normalized values of four independent experiments plus standard error of the mean (SEM). Numbers in or on columns indicate the number of analyzed animals (ntotal = 712). ***: p<0.001; Kruskal-Wallis test plus Dunn's Multiple Comparison Test; Mann Whitney test (comparison of vector at 15°C and 25°C). Equal optical settings, scale bar 200 µm.(i): RNAi; L4440: empty vector control. (PDF) [file pgen.1003346.s005.pdf]

Figure S6:

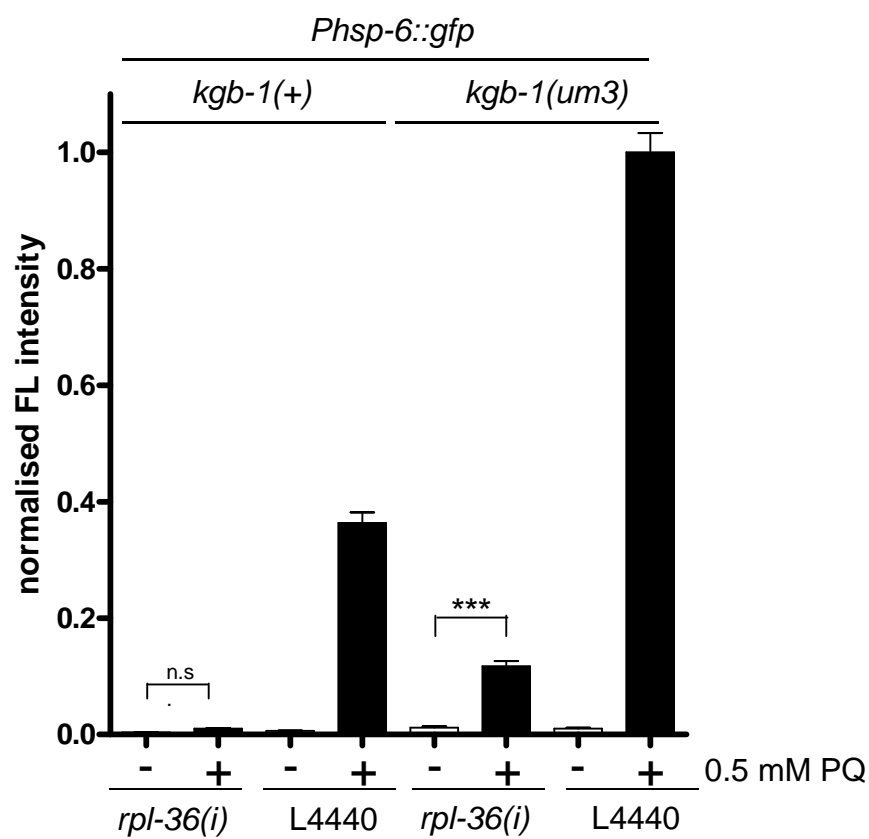

Supplement: Figure S6 — The cSADDs inhibit paraquat mediated signaling to hsp-6. The loss of the hsp-6::gfp induction in rpl-36(RNAi) is suppressed by mutant kgb-1(um3), indicating a KGB-1 mediated repression of UPRmt by cSADDs. rpl-36(RNAi) was shown to be sufficient to induce cSADDs [3]. Columns represent normalized values plus standard error of the mean (SEM). ***: p<0.001; Kruskal-Wallis test plus Dunn's Multiple Comparison Test. (i): RNAi; L4440: empty vector control; (+): wild-type allele. (PDF) [file pgen.1003346.s006.pdf]
