# Supplementary material for: Surveillance-Activated Defenses Block the ROS–Induced Mitochondrial Unfolded Protein Response
Source: PLoS Genet. 2013 Mar 14;9(3):e1003346. doi: 10.1371/journal.pgen.1003346 (PMC3597513; doi:10.1371/journal.pgen.1003346)
Supplement: Table S1 — Knockdown of 36 of 55 screening positives were shown by Melo and Ruvkun, 2012 [3] to evoke aversion behavior. This table displays the subset of our screening positives which were recently shown to trigger aversion [3]. (DOCX) [file pgen.1003346.s007.docx]

### Table S1: Knockdown of 36 of 55 screening positives were shown to evoke aversion behavior by Melo and Ruvkun, 2012 [1].

| **Gene** | **Brief description** |
| --- | --- |
| *act‑3* | actin |
| *apm‑1* | Adaptor complexes medium subunit |
| *cct‑1* | cytosolic chaperonin, subunit |
| *cct‑2* | cytosolic chaperonin, subunit |
| *cct‑4* | cytosolic chaperonin, subunit |
| *elt‑2* | GATA‑4/5/6 transcription factor |
| *imb‑3* | nuclear transport factor |
| *imb‑5* | nuclear transport factor |
| *pan‑1* | predicted transmembrane protein |
| *pas‑4* | 20S proteasome, regulatory subunit |
| *pas‑7* | 20S proteasome, regulatory subunit |
| *phi‑2* | eIF‑4A |
| *phi‑4* | mRNA splicing factor |
| *rpl‑14* | large ribosomal subunit protein |
| *rpl‑17* | large ribosomal subunit protein |
| *rpl‑18* | large ribosomal subunit protein |
| *rpl‑22* | large ribosomal subunit protein |
| *rpl‑23* | large ribosomal subunit protein |
| *rpl‑30* | large ribosomal subunit protein |
| *rpl‑31* | large ribosomal subunit protein |
| *rpl‑35* | large ribosomal subunit protein |
| *rpl‑36* | large ribosomal subunit protein |
| *rpl‑41* | large ribosomal subunit protein |
| *rpn‑7* | 19S proteasome, regulatory subunit |
| *rps‑14* | small ribosomal subunit protein |
| *rps‑17* | small ribosomal subunit protein |
| *rps‑26* | small ribosomal subunit protein |
| *rps‑27* | small ribosomal subunit protein |
| *rps‑8* | small ribosomal subunit protein |
| *snr‑1* | small nuclear ribonucleoprotein/U1snRNP |
| *snr‑2* | small nuclear ribonucleoprotein/U1snRNP |
| *snr‑6* | small nuclear ribonucleoprotein/U1snRNP |
| *vha‑1* | Vacuolar H+‑ATPase subunit |
| *vha‑2* | Vacuolar H+‑ATPase subunit |
| Y39B6A.42 |  |
| Y65B4A.6 | predicted ATP‑dependent RNA helicase FAL1 |
