# Supplementary material for: Correction: Surveillance-Activated Defenses Block the ROS-Induced Mitochondrial Unfolded Protein Response
Source: PLoS Genet. 2016 Oct 7;12(10):e1006377. doi: 10.1371/journal.pgen.1006377 (PMC5065125; doi:10.1371/journal.pgen.1006377)
Supplement: S1 File — (DOCX) [file pgen.1006377.s002.docx]

# Surveillance activated defenses block the ROS induced mitochondrial unfolded protein response

**Eva D. Runkel^1,2^, Shu Liu^2,5^, Ralf Baumeister^1-4^* and Ekkehard Schulze^2,4^**

^1^ Spemann Graduate School of Biology and Medicine, Albert-Ludwigs-University of Freiburg, Freiburg, Germany

^2^ Bioinformatics and Molecular Genetics (Faculty of Biology), Albert-Ludwigs-University of Freiburg, Freiburg, Germany

^3^ Center for Biochemistry and Molecular Cell Research (Faculty of Medicine), Albert-Ludwigs-University of Freiburg, Freiburg, Germany

^4^ Centre for Biological Signaling Studies (BIOSS), Albert-Ludwigs-University of Freiburg, Freiburg, Germany

^5^ current address: Deutsches Zentrum für Neurodegenerative Erkrankungen e.V. (DZNE), Bonn, Germany

* Corresponding author

Schaenzlestr. 1

D-79104 Freiburg

Tel. +49-761-203 8350

Fax +49-761-203 8351

Email: baumeister@celegans.de

## ABSTRACT

Disturbance of cellular functions results in the activation of stress signaling pathways that aim at restoring homeostasis. We performed a genome wide screen to identify components of the signal transduction of the mitochondrial unfolded protein response (UPR^mt^) to a nuclear chaperone promoter. We used the ROS generating complex I inhibitor paraquat to induce the UPR^mt^, and we employed RNAi exposure post-embryonically to allow testing genes whose knockdown results in embryonic lethality. We identified 53 novel regulators of the ROS induced UPR^mt^. Activation of the UPR^mt^, but not of other stress signaling pathways, failed when homeostasis of basic cellular mechanisms such as translation and protein transport were impaired. These mechanisms are monitored by a recently discovered surveillance system that interprets interruption of these processes as pathogen attack, and depends on signaling through the JNK-like MAP-kinase KGB‑1. Mutation of *kgb‑1* abrogated the inhibition of ROS induced UPR^mt^, suggesting that surveillance activated defenses specifically inhibit the UPR^mt^, but do not compromise activation of the heat shock response, the UPR of the endoplasmic reticulum, or the SKN‑1/Nrf2 mediated response to cytosolic stress.

## AUTHORS' SUMMARY

Cellular respiration takes place in the mitochondria. Reactive oxygen species (ROS) are a damaging byproduct of cellular respiration. At adverse conditions, when the load of ROS becomes critical for mitochondrial function, a stress response pathway, the unfolded protein response of the mitochondria (UPR^mt^) is triggered. This can be monitored by the activation of the *hsp‑6* gene. We used the model organism *Caenorhabditis elegans* to screen for genes required for the activation of *hsp‑6* and found 53 novel candidates. Surprisingly, most of the genes we identified serve basic cellular functions and are no plausible candidates for regulatory functions. However, this group of genes was recently shown to trigger a cellular surveillance mediated stress response, sensing pathogen invasion and toxin attack, and forcing the animals to escape from environmental hazards. This mechanism partially requires signaling through the kinase KGB‑1. In worms, in which KGB‑1 was inactivated, UPR^mt^ was not longer interrupted by downregulation of the cellular surveillance activating genes. We suggest this surveillance system as a regulator of the UPR^mt^ that prevents its activation when pathogen attack is sensed.

## INTRODUCTION

In order to survive, organisms have to deal with an adverse environment either by avoiding unfavorable or toxic conditions, or by dealing with the consequences of such exposure. The nematode *C. elegans* for this purpose has developed a number of survival strategies. First, the sensory capabilities of this soil-dwelling animal enable the detection of probably hundreds of adverse mechanical, thermal and chemical stimuli. These neurons are wired to interneurons that serve as a neuronal processor with analytical power, which in turn couples to a motor response to search for or avoid certain environmental conditions. Second, mechanisms have been established in *C. elegans* to prevent uptake, to inactivate detrimental chemicals, or to repair the consequences of toxin exposure [1-4].

As part of an avoidance strategy to minimize future encounter of a toxin, it was recently reported that *C. elegans* surveys pathways typically disrupted by pathogens or toxins to engage in defenses. Experimental inactivation of genes in these pathways was sufficient to stimulate an aversion behavior in which the animals avoid normally attractive bacteria [3]. In this study, a large number of genes were found suggesting that this surveillance system (cSADDs) monitors the activity of core cellular components, including translation, energy metabolism, and protein degradation, and triggers food aversion, innate immunity and detoxification defenses upon detection of perturbations.

Unfolded protein responses (UPRs) are evoked when unfolded or misfolded proteins exceed the chaperone folding capacity of the cell. In eukaryotes, individual UPR pathways have evolved for distinct subcellular compartments, such as the endoplasmic reticulum (ER) or the cytosol (for review, see [5,6]). To restore protein homeostasis, the UPRs signal from the stressed subcellular compartment to the nucleus and initiate an upregulation of a discrete set of compensatory genes, among them compartment-specific chaperones (for review, see [7,8]). In the nematode *C. elegans,* reporter gene fusions of the promoters of the respective chaperones have been applied to study the UPR pathways [9].

The cytosolic UPR, also known as heat shock response, is initiated by stress interfering with the cytosolic protein folding environment (heat, e.g.) and activates genes including the cytosolic chaperone gene *hsp‑16.2* [10,11]. In the endoplasmic reticulum (ER), protein folding stress can be experimentally evoked by the administration of tunicamycin, an inhibitor of protein glycosylation [12], that triggers an unfolded protein response (UPR^ER^) to upregulate the transcription of the ER-specific chaperone gene *hsp‑4* [13] and results, among others, in a general blockade of translation.

Cytosolic oxidative stress elicits responses that in higher eukaryotes activate the phase II detoxification system that is triggered by the transcription factor SKN‑1/Nrf2. In *C. elegans*, this pathway cross-talks with the DAF‑2/Insulin/IGF receptor pathway, signaling to its main effector, the transcription factor DAF‑16/FOXO [14]. A number of genes have been identified that are differentially regulated by SKN‑1, DAF‑16, or a combination of both ([15-20], for review see [21]).

Beside the UPR of the cytosol and the ER, more recently an unfolded protein response specific to mitochondria has been described ([22-25], for review see [26,27]). The unfolded protein response of the mitochondria (UPR^mt^) is initiated by several modes of mitochondrial stress and activates the expression of nuclear genes, among them *hsp‑6* and *hsp‑60* encoding mitochondrial chaperones [22]. Many of the described UPR^mt^ inducing stressors interfere directly with the mitochondrial protein folding environment: Inducing stress signals include the downregulation of the mitochondrial chaperone genes *hsp‑6* and *hsp‑60*, or knockdown of *spg‑7* encoding a mitochondrial protease [22], or genes encoding components of the ETC which function in a cell non-autonomous way [28]. A temperature-sensitive mutation, *zc32*, whose corresponding gene is still enigmatic, was phenotypically characterized and shown to conditionally activate the UPR^mt^ [23]. Several molecular components of the UPR^mt^ pathway have been proposed and suggested a mechanistic model (for review, see [26,27]) in which, as a first step, accumulated unfolded or misfolded proteins are cleaved by the ClpP protease in the mitochondrial matrix [24]. Partly through the HAF‑1 ABC transporter, the bZip transcription factor ATFS‑1 is activated, whose nuclear targeting in turn directly induces the transcription of the mitochondrial chaperone genes *hsp‑6* and *hsp‑60* [25,29]. The homeobox transcription factor DVE‑1 and the ubiquitin-like protein UBL‑5 are also part of this UPR^mt^ model and induce, independently of ATFS‑1, mitochondrial chaperone expression upon peptide efflux from HAF‑1 [23-25,30,31]. Recently, a much simpler mechanism was suggested by the same researchers. Under non-stress conditions, *atfs‑1* mRNA in the cytosol generates a transcription factor which, by default, is transported via the TIM-TOM import complexes into the mitochondria and there is proteolytically inactivated. Stress that alters the mitochondrial membrane potential blocks protein import of ATFS‑1, resulting in its cytosolic accumulation and subsequent nuclear transport, where it can activate *hsp‑6* and *hsp‑60* genes [29].

Mutations in proteins of the mitochondrial electron transport chain (ETC) typically distort electron transfer to oxygen and, thus, generate reactive oxygen species (ROS). Recently, it was suggested that, in addition to recognizing protein misfolding stress, ROS in a parallel pathway may generate a signal to downregulate translation initiation via the GCN‑2 dependent phosphorylation of eIF2α [32]. Thus, in analogy to the UPR^ER^, it was proposed that activating the UPR^mt^ has two consequences: Downregulation of translation, and selective activation of expression of chaperone genes.

Paraquat is a non-selective contact herbicide that in experimental research is frequently used to provoke the generation of reactive oxygen species in the cell, since it accepts electrons in the electron transport chain (ETC) at the inner mitochondrial membrane and transfers them to molecular oxygen, generating the superoxide anion [33-36]. Paraquat administration, among others, induces the mitochondrial manganese superoxide dismutase gene *sod‑3* [22], which is known to respond to increased ROS [37], and also the UPR^mt^ responsive gene *hsp‑60* [22]. The onset of the UPR^mt^ reporter upon paraquat-mediated accumulation of ROS may be due to consecutive protein damage, such as irreversible protein carbonylations [38]. It was shown in recent years that a moderate elevation of ROS generated in the mitochondria, such as in a loss of function mutant of the ETC component ISP‑1 [34,39], leads to an increase in lifespan [34,40]. This effect can also be mimicked by low concentrations of paraquat [34,40,41]. Thus, administration of paraquat/ROS may have either detrimental or beneficial consequences for a cell or an organism.

Here, we investigate the retrograde signaling to the *hsp‑6* promoter initiated by an increase in mitochondrial ROS, which we trigger by low doses of paraquat. Genome-scaled RNAi screening revealed, among others, ATFS‑1 as essential for the retrograde mitochondrial stress response to ROS, similar to its role in UPR^mt^. We also found that HAF‑1 is dispensable for the paraquat induced signaling, suggesting that a peptide efflux via HAF‑1 is not required after ROS induced mitochondrial stress to induce the *hsp‑6* promoter. We identified 53 additional genes whose downregulation prevented the activation of *hsp‑6*. 89% of them were previously shown to encode components of cellular surveillance monitored pathways, or were found in protein complexes involved in surveillance monitored pathways (cSADDs). We postulate that cellular surveillance serves as a master regulator, activation of which inhibits the onset of the paraquat induced UPR^mt^. Our model suggests that *C. elegans* uses decisions at several levels to protect itself from external and internal stress.

## RESULTS

### Paraquat induces *hsp‑6* reporter expression

Expression of the mitochondrial chaperone gene *hsp‑6* is induced upon treatment of *C. elegans* with paraquat (Fig. 1A), which has been considered to activate oxidative stress [34,39,42,43] and the mitochondrial unfolded protein response (UPR^mt^) [22]. We devised a new protocol for paraquat administration which allowed the detection of essential embryonic genes involved in the UPR^mt^, which could not have been found in previous screens to identify components of the UPR^mt^. In this protocol L3 stage animals were cultured with paraquat for two days. To monitor stress resistance pathways we used a previously described *hsp‑6::gfp* reporter strain which carries the *zcIs13* transgene containing about 1.7 kb of the 5' flanking region and the first 10 codons of *hsp‑6* fused to GFP [22]. We performed a concentration series and observed that *hsp‑6::gfp* induction peaked around 0.5 to 1.7 mM and faded with increasing concentrations of paraquat correlating with increased toxicity (Fig. S1). To lower the possible impact of toxicity, we performed subsequent experiments using the lower concentration of 0.5 mM paraquat. This induced the *hsp‑6* reporter 41‑fold (Fig. 1B). Visual inspection by stereomicroscopy revealed that GFP expression started one day after paraquat exposure.

In addition to the expression of *hsp‑6*, activated UPR^mt^ can also be monitored by GFP expression from the promoter of *hsp‑60* [22]. Comparing the fluorescence intensity of both reporters after paraquat exposure showed that at 0.5 mM *hsp‑6::gfp* was induced significantly while *hsp‑60::gfp* was not (Fig. S2). At a paraquat concentration of 2.0 mM, both reporters were significantly induced, as published [22], *hsp‑6::gfp* induction, however, was twenty times stronger (Fig. S2). We concluded that *hsp‑6::gfp* is the more sensitive reporter for monitoring paraquat exposure, and, thus, performed subsequent experiments with this reporter.

### Paraquat induces *hsp‑6* reporter expression independent of HAF‑1, but requires ATFS‑1

The gene *haf‑1* encodes a mitochondrial inner-membrane localized ABC transporter considered necessary for mitochondrial peptide release to activate the transcription factor ATFS‑1, resulting in its nuclear translocation and activation of *hsp‑6*. HAF‑1 was suggested to be an essential upstream component of the UPR^mt^, since it was reported that in a *haf‑1(ok705)* deletion mutant neither the *hsp‑6* nor the *hsp‑60* reporters were induced by RNAi with *spg‑7* or by the uncharacterized *zc32* mutation, which is a standard inducer of UPR^mt^ [25]. In the presence of 0.5 mM paraquat we observed that *haf‑1* was dispensable for the activation of *hsp‑6::gfp* (Fig. 2). Moreover, we observed a hyper-activation of *hsp‑6::gfp* indicating that, at this condition, loss of *haf‑1* may further induce rather than block *hsp‑6* expression (Fig. 2A). In contrast, ATFS‑1, which integrates UPR^mt^ signaling at the *hsp‑6* promoter [25,32], was required for paraquat induced *hsp‑6* expression. Knockdown of *atfs‑1* by RNAi abolishes the inducibility of the reporter completely (Fig. 2). These data suggest that low doses of paraquat mediated the activation of *hsp‑6::gfp* through ATFS‑1, but do not require HAF‑1.

### ETC impairment by ROS activates *hsp‑6::gfp*

The UPR^mt^ was so far primarily investigated with stressors that seem to cause unfolded protein stress by directly interfering with mitochondrial proteostasis (such as the knockdown of mitochondrial chaperones or the inactivation of mitochondrial proteases [22-24,27]). Paraquat, in contrast, is a compound primarily known to cause oxidative stress impairing the ETC [42-46]. We wondered whether the induction of *hsp‑6::gfp* is specific for paraquat or whether also other conditions known to increase mitochondrial ROS can activate the reporter. We exposed the *hsp‑6* reporter strain from early L3 stage on for two days to 0.25 µM rotenone, which targets the ubiquinone of complex I, or to 0.25 µM antimycin A, which prevents electron transfer from coenzyme Q to cytochrome C. Both substances have been shown to increase the amount of ROS [33,36]. As with paraquat, both toxins caused an induction of the *hsp‑6* reporter (Fig. 3A) being in line with the idea that an increase in mitochondrial ROS induces *hsp‑6*.

Genetic interference with the ETC by the introduction of a missense mutation in the *isp‑1* allele *qm150* increases mitochondrial superoxide [34]. Supporting our previous findings, *hsp‑6::gfp* was induced as well. GFP was constitutively expressed in the *isp‑1(qm150)* mutant through all developmental stages and adulthood (Fig. 3A). A similar result was observed for the *mev‑1(kn1)* mutant, which carries a mutation in the cytochrome b of the mitochondrial respiratory chain complex II (data not shown).

We next wondered whether a compound causing oxidative stress not obviously linked to mitochondrial or ETC dysfunction can also activate *hsp‑6::gfp*. The neurotoxin acrylamide triggers cytosolic phase II antioxidant responses in a SKN‑1 dependent manner [47,48]. Its mode of ROS production has not been associated with mitochondrial function [49]. We cultured early L3 larvae of the *hsp‑6* reporter strain for two days with 2.1 mM acrylamide, and observed only a slight 1.5‑fold induction of the *hsp‑6* reporter, whereas paraquat induced the *hsp‑6* reporter 25 times more effectively (Fig. 3B). The same acrylamide concentration, however, sufficed to activate the phase II response, monitored by the induction of *gst‑4::gfp*. Our results suggest that *hsp‑6::gfp* responds to both mutants and substances that are considered to increase mitochondrial ROS.

### The ROS scavenger NAC reduces paraquat mediated *hsp‑6* induction

To analyze whether the increase in mitochondrial ROS is causative for the induction of the *hsp‑6* reporter, we compared the response of paraquat treated *hsp‑6* reporter animals with those in which paraquat treatment was paired with the addition of 10 mM of the ROS scavenger N-acetyl-L-cysteine (NAC) [50,51]. We observed a 75 % reduction in the intensity of *hsp‑6::gfp* fluorescence in the presence of NAC (Fig. 1C). This suggests that paraquat induced ROS is coupled to *hsp‑6::gfp* induction. With this experiment we cannot distinguish whether the UPR^mt^ is induced directly by the increased level of ROS or through a secondary protein damage caused by ROS. In the latter case, NAC treatment may also ultimately prevent protein misfolding by scavenging ROS and thus reducing *hsp‑6::gfp* induction. Since the ROS scavenger NAC reduced *hsp‑6::gfp,* rather than increased the induction, we consider it unlikely that paraquat induced ROS serves as a signal rather than a toxin, as has been proposed recently [32]. We conclude that ROS increase is a primary causative element in the induction of *hsp‑6::gfp*, which, however, may also evoke the response of *hsp‑6* through an increase in mitochondrial unfolded proteins.

### Paraquat treatment alters mitochondrial morphology

It is possible that 0.5 mM paraquat increases ROS production and signaling to *hsp‑6* directly or by affecting the integrity or functions of mitochondrial proteins. An effect of paraquat (0.1 mM) on protein oxidative damage has been shown, even though the abundance of mitochondria was not affected [34]. An altered mitochondrial morphology has been used as an argument for high levels of protein stress in the organelle [24]. Mitotracker is a compound that was used before in experiments to stain mitochondrial membranes [2,16,17]. Even at the low concentration of 0.5 mM paraquat, we found that our treatment resulted in substantial structural alterations of the hypodermal mitochondrial membrane (Fig. 4A). We confirm in this experiment that 0.5 mM paraquat treatment alters mitochondrial structure, even though the general constitution and the morphology of animals is barely affected, except for a slight but obvious reduction in the body size of treated animals (see also Fig. S1).

### Paraquat does not induce unfolded protein responses in the ER or cytosol

Next, we investigated whether 0.5 mM paraquat induced stress responses in cellular compartments other than the mitochondria. Thus, we tested whether reporters of the unfolded protein response of the ER (*hsp‑4::gfp*) [10] or of the cytosol (*hsp‑16.2::gfp*) [9] also responded to paraquat treatment. We applied paraquat at early L3 stage and analyzed reporter fluorescence two days later. While both reporters were induced by their respective specific triggers tunicamycin and heat stress, no significant induction was observed with paraquat (Fig. 4B,C). We conclude that 0.5 mM paraquat does not activate the unfolded protein response in the ER or in the cytosol.

### Induction of *hsp‑6* by paraquat is independent of key regulators of other ROS stress responses

While paraquat does not activate cytosolic UPR, it is known to activate the cytosolic oxidative stress reporter *gst‑4::gfp* [52]. In *C. elegans*, several key regulators of cytosolic oxidative stress responses have been described before. The transcription factors SKN‑1 and DAF‑16 are crucial for the induction of the phase II oxidative stress response and the defense against oxidative damage, respectively [53,54]. Hypoxia inhibits respiration and activates HIF‑1 by elevating the levels of ROS [40]. Therefore, expression of *hsp‑6* by paraquat could be dependent on these regulators. We therefore tested paraquat-induced expression of *hsp‑6::gfp* in loss-of-function mutants of *skn‑1(zu67)*, *daf‑16(mu86)* and *hif‑1(ia4)*. We found that none of the mutants prevented the induction of *hsp‑6* upon 0.5 mM paraquat exposure (Fig. 5). This suggests that the response to paraquat triggers a pathway that does not require the transcription factors SKN‑1, DAF‑16 and HIF‑1, and probably also not the pathways in which these factors are effectors namely the cytosolic stress response, insulin signaling, and the heat shock response.

### A genome-scaled RNAi screen identifies 54 genes required for paraquat triggered *hsp‑6::gfp* induction

To identify essential components of the paraquat mediated induction of *hsp‑6::gfp*, we screened the ORFeome RNAi library (Open Biosystems) [55] for suppressors. Synchronized L1 larvae were allowed to develop by feeding on the respective RNAi bacteria for one day. Then, they were exposed to paraquat in order to bypass the paraquat-hypersensitive L1/early L2 stage and to benefit from the enhanced paraquat inducibility of the *hsp‑6* reporter in the L3 larval stage. After two days of incubation with paraquat we screened for worms that had failed to induce the *hsp‑6* reporter assuming that the respective RNAi clone downregulated a factor essential for *hsp‑6* induction (Fig. S3).

We confirmed 54 genes whose knockdown led to an evident impairment of *hsp‑6::gfp* induction (Table 1). The majority of these also showed morphological, behavioral or developmental abnormalities, among them impaired movement and developmental delay or arrest, which in each case appeared independent of paraquat administration.

Based on GO term analysis [56] the genes of all screening positives were assigned to functional groups. We found two subunits of a vacuolar H^+^ ATPase (Functional Group: ATP synthesis coupled proton transport), several proteasomal regulatory subunits (Functional Group: Cellular protein catabolic process (Proteolysis)), and several subunits of the cytosolic chaperonin complex, the orthologue of human TRiC/CCT (TCP‑1 Ring Complex) (Functional Group: Cellular protein metabolic process (Protein folding)). We also detected several genes encoding proteins involved in intracellular protein transport, including two nuclear transport factors (Functional Group: Intracellular protein transport). Furthermore, the screen revealed three genes encoding small nuclear ribonucleoproteins (Functional Group: mRNA splicing). A large group, 26 genes, encode proteins of both ribosomal subunits (20 out of 54 screening positives) and additional genes (6 out of 54 screening positives) whose products have been associated with the translation of proteins (Functional Group: Protein translation). The mechanisms through which knockdown of these genes prevents *hsp‑6* reporter induction is not an attenuation of the general translation, since the inhibitory effect could not be mimicked if translation was attenuated by other means (Fig. S4). Two genes (Functional Group: Regulation of transcription, DNA dependent) encode transcription factors, one being the GATA type transcription factor ELT‑2, which is required for intestinal cell differentiation and maintenance [57,58], and the other being ATFS‑1, the bZip transcription factor involved in UPR^mt^ [25]. The detection of the latter in the unbiased screen confirmed our observation that ATFS‑1, in line with its previously described role in UPR^mt^ signaling [25,32], is also involved in the *hsp‑6::gfp* induction by paraquat (see Fig. 2). One functional group was assigned to a gene whose product is putatively involved in signaling (Functional Group: Signaling). Seven genes were not clustered in groups (Table 1). We quantified the *hsp‑6* reporter induction by paraquat for two screening positives, *rpl‑36* and *atfs‑1.*RNAi against each gene significantly prevented *hsp‑6::gfp* induction (Fig. 6).

Except for ATFS‑1 none of the 54 screening positives had been implicated in the UPR^mt^ before. In order to test whether we found so far not described genes in the UPR^mt^ pathway, we performed RNAi against all in the temperature-sensitive mutant strain SJ52 [*zc32;* *Phsp‑60::gfp*] used in a previous UPR^mt^ screen. Since many of our screening positive RNAis caused larval arrest, the UPR^mt^ screening protocol used by these authors was modified [23,24]. We added synchronized L1 larvae to the respective RNAi plates, shifted the plates from 15°C to the restrictive temperature of 25°C when worms had developed to L4 larvae or young adults, and analyzed GFP fluorescence after two additional days. This protocol allowed the analysis of the role of our embryonic or larval lethal screening positives according to the UPR^mt^ model. 29 candidates interfered with the activation of the *hsp‑60::gfp* reporter in the *zc32* mutant, among them *atfs‑1* which we considered as confirmation of the quality of our protocol (Table 1), 24 candidates did not obviously alter *hsp‑60::gfp* expression in the background of *zc32*. We quantified GFP intensity in *zc32* mutant animals expressing *hsp‑60::gfp* which were treated with *atfs‑1*and *rpl-36* RNAi. Confirming a previous report, knockdown of *atfs‑1* attenuated *hsp‑60* reporter induction [25] (Fig. S5). In contrast, downregulation of *rpl‑36*, which encodes a protein of the large ribosomal subunit, rather increased GFP expression of the *hsp‑60* reporter compared to the vector control (Fig. S5). These results confirm that *atfs-1* is required, whereas *rpl-36* may be dispensable for the *zc32* triggered mitochondrial stress response (Table 1). Since the identity of the *zc32* mutation is still enigmatic, it is currently not possible to interpret the difference obtained in both experimental paradigms.

### ATFS‑1 and RPL‑36 are also required for *isp‑1(qm150)* mediated induction of *hsp‑6::gfp*

Beside paraquat other ROS-generating compounds and ETC mutants induce *hsp‑6::gfp* (see Fig. 3A). Here we tested whether the block of *hsp‑6::gfp* induction is either specific for paraquat or if it would also block *hsp‑6::gfp* induction resulting from the ETC mutant *isp‑1(qm150)*, which is a generator of mitochondrial superoxide [34]. RNAi specifically impairing paraquat uptake or metabolism would not block the *isp‑1(qm150)* mediated signaling. We tested RNAi of *atfs‑1*, which is known to be part of the UPR^mt^ and *rpl‑36*, which is not essential for the *zc32*, but for the paraquat-triggered *hsp‑6::gfp* induction. GFP expressing L1 larvae were placed on the respective RNAi plates and grown until adulthood. Analyses in adulthood were possible since RNAi against both genes did not cause larval arrest. During larval growth, GFP was continuously expressed on control plates. On the RNAi plates however, GFP fluorescence was strongly reduced. Downregulation of all three screening positives during postembryonic stages of *isp‑1(qm150)* abrogated the induction of the *hsp‑6* reporter (Fig. 7).

We conclude from these data that none of the tested screening positives affects paraquat metabolism but rather mechanistic steps in the signaling cascade. However, alternatively it is also possible that these RNAis relieve the worms of ROS stress and thereby prevent the induction of *hsp‑6::gfp*.

### Knockdown of screening positive genes increases paraquat sensitivity

The expected function of a stress signaling pathway is to trigger a protective response. Screening positives may emerge for two different reasons: Either a specific stress signaling pathway could be blocked, or, alternatively, the level of stress could be reduced. In order to distinguish between these two alternatives we reasoned that a relief of stress would lead to paraquat hyposensitivity or resistance, whereas the disruption of a protective function would lead to an increased sensitivity to paraquat. We tested RNAi with two exemplary screening positives (*atfs‑1*, *rpl‑36*)in a paraquat toxicity assay during larval development. Synchronized L1 larvae were raised on the respective RNAi plates containing 0.4 mM paraquat, and the number of animals on each plate that reached adulthood at day 5 was counted. Without paraquat, all worms developed to become adults, except for *rpl‑36* (RNAi) of which 1.5 % did not reach adulthood within this time. Following paraquat exposure, about 50 % of the controls became adults until day 5. In contrast, none of the animals subjected to RNAi against *atfs‑1* and *rpl‑36* respectively reached adulthood in this time window. This data indicate that RNAi against *atfs‑1* and *rpl‑36*increased, rather than decreased, paraquat sensitivity (Fig. 8). Thus, for these two exemplary screening positives a relief of stress scenario can be ruled out. Furthermore, we conclude that the established UPR^mt^ component *atfs‑1* contributes to a protective response in line with a previous report [32].

### Most genes required for ROS-dependent *hsp‑6* induction selectively affect the response to mitochondrial stress

We noticed that none of our screening positives encodes a mitochondrial protein. Therefore it was important to investigate, if they function either in general stress responses or have a specific role in the mitochondrial stress response. Therefore we examined their putative function in the phase II oxidative stress response, the cytosolic unfolded protein response (heat shock response) and the unfolded protein response of the ER (UPR^ER^). All 54 screening positives were assessed qualitatively by visual inspection under the dissecting microscope to test whether the respective RNAi knockdown abrogated GFP fluorescence of the reporters tested. To get more detailed insights we quantified two candidate screening positives (*atfs‑1*, *rpl‑36*) for each stress response. ATFS‑1 was chosen as a known UPR^mt^ pathway component and *rpl‑36* RNAi enhanced *zc32* triggered mitochondrial stress signaling but abolished paraquat mediated induction of the *hsp‑6* reporter.

Acrylamide induces a SKN‑1 dependent induction of *gst‑4::gfp* [47,52,59]. RNAi knockdown of none of the 54 candidates blocked *gst‑4* expression in response to 2.1 mM acrylamide (Table 1). Quantification of two screening positives revealed that *gst‑4::gfp* fluorescence was not suppressed by *rpl‑36* and *atfs‑1* RNAi, suggesting that the inactivation of these genes does not interfere with the class II response (Fig. 9A).

We noticed that RNAi of *cct‑1*, *cct‑5*, *pas‑4,* and *pas‑7* already resulted in *gst‑4* expression in the absence of acrylamide, confirming a previous report [48] (Table 1). Thus, for those four candidates that affect protein folding and turnover, we could not exclude that such constitutive activation of the class II detoxification system might reduce the ROS burden after paraquat administration. This would render the worms more resistant to paraquat, and could explain why *hsp‑6* is not induced in those four experiments. While the *cct‑1*/*‑*5 RNAi mediated induction of *gst‑4* appeared to be independent of SKN-1, knockdown of the proteasomal subunit mitigates *gst‑4* expression via SKN-1 [48]. We anticipated, therefore, that such an indirect effect would be SKN‑1 dependent, at least in case of RNAi against a proteasomal subunit gene. Therefore, we tested paraquat mediated *hsp‑6* induction in *skn‑1(zu67)* mutant animals after RNAi with *pas‑4,* and *pas‑7*. Loss of function of SKN‑1 did not reconstitute the paraquat mediated *hsp‑6* induction, which argues against such an indirect effect of the SKN-1 activating RNAi experiments. However, a SKN-1 independent relief of stress cannot be excluded.

Next, we tested whether the screening positives crosstalk with the cytosolic unfolded protein response. We heat-shocked L4 staged *hsp‑16.2::gfp* reporter worms for 4h at 34°C and observed fluorescence one day later. Qualitative assessment of GFP fluorescence revealed no obvious impairment in any of the RNAi experiments (Table 1). Knockdown of *rpl‑36* and *atfs‑1*, the two factors affecting the UPR^mt^ response, did not prevent, but significantly decreased *hsp‑16.2* induction to 34% and 58%, respectively (Fig. 9B). This suggests that some crosstalk between the UPR^mt^ and the heat shock responses exists, or that these genes have dual roles in both pathways. This would make sense, since noxious heat will also result in denaturation of mitochondrial proteins, which may also increase mitochondrial ROS production. Since most of the factors involved in UPR^mt^ are cytosolic signaling components [25], the same proteins could also help in activating the cytoplasmic heat shock response.

Next, a possible role of the screening positives in the induction of the unfolded protein response of the endoplasmic reticulum (UPR^ER^) was tested. UPR^ER^ was triggered by incubation with 7.2 µM tunicamycin and monitored using the *hsp‑4::gfp* reporter [9]. We found three screening positives (*vha‑1*, *snap‑1*, and *sec‑23*) whose knockdown induced the *hsp‑4* reporter already in the absence of tunicamycin implicating that the loss of expression of those genes induces ER stress constitutively. All three candidates play a role in intracellular protein transport. Visual inspection revealed that none of the RNAi treated screening positives prevented or strongly reduced *hsp‑4::gfp* induction (Table 1). Quantification of *atfs‑1* and *rpl‑36* showed that RNAi with *atfs‑1* did not affect induction significantly, whereas *rpl‑36* reduced the induction to 49 %, which proved to be significant (Fig. 9C). Thus, it may be possible that affecting the balance of ribosomal protein expression interferes with the induction of unfolded protein responses in both ER and mitochondria.

### cSADDs suppresses the response of *hsp‑6* to paraquat

We noticed that many of the screening positives we had identified are genes also identified in a recent publication by Melo *et al.* (2012). There, the authors report a cellular surveillance system, which they call cSADDS (cellular surveillance activated detoxification and defenses) that monitors basic cellular functions and, if compromised, [generate](http://dict.leo.org/ende?lp=ende&p=L3YAA&search=generate&trestr=0x8002)s specific behavioral, immune, and detoxification responses, respectively [3]. Downregulation of 36 of the 54 genes (65 %) identified in our screen was identified to induce the cSADDs, including food aversion behavior ([3], Table S1). In addition, of the remaining 19 genes twelve encode proteins belonging to either functional protein classes or protein complexes which activate the cellular surveillance system upon distortion [3]. Thus, in total 89 % of the screening positives encode proteins belonging to processes or complexes that are monitored by the surveillance system.

Given that the cellular surveillance system is monitoring life-threatening conditions, such as toxin or pathogen exposure, we hypothesized that cSADDs may inhibit the onset of other stress responses that are evoked by milder, not life-threatening stresses, like the concentrations we have chosen for paraquat administration to induce the UPR^mt^. Signaling from cellular surveillance partially requires the activity of a JNK signaling cascade, in which KGB‑1 is an essential component. We hypothesized that interfering with the signaling of cSADDs by a mutation in *kgb‑1* should at least partially release its inhibitory impact of the UPR^mt^. To test this idea, we crossed the *hsp‑6* reporter strain with the *kgb‑1(um3)* mutant, which has been shown to partially suppress the surveillance mediated food avoidance [3]. Then, in the presence of 0.5 mM paraquat, as used in our screening protocol, *kgb‑1(um3)*; *hsp‑6::gfp* worms were grown on *elt-2(RNAi)* bacteria, knockdown of which triggers cSADDs mediated aversion [3]. Whereas downregulating *elt‑2* in *kgb‑1*(+) control strains eliminated the GFP induction as reported, the introduction of the *kgb‑1(um3)* mutation released this inhibitory effect to some extent (Fig. 10A). We observed the same recovery of *hsp‑*6::gfp induction in the *kgb‑1(um3)* mutant when worms were grown on *rpl‑36* RNAi (Fig. S6). We conclude that *elt‑2* and *rpl‑*36, and probably the other 46 genes whose RNAi activated cSADDs*,* contribute to cSADDs mediated inhibition of the mitochondrial stress response.

In line with this idea, loss of *kgb‑1* would not affect the inhibitory effect of those screening positives that do not do not evoke cSADDs. ATFS‑1, the transcription factor controlling *hsp‑6* activation [25] has not been detected as an activator of cSADDs [3]. We therefore first tested whether RNAi with *atfs-1* triggers the aversion phenotype, that served as a readout for cSADDs [3]. RNAi with *atfs-1* did not induced food aversion, whereas RNAi of *elt‑2*, used as a control as described [3], did (Table 2). Next, we tested whether in a *kgb‑1* mutant paraquat triggered *hsp‑6::gfp* induction is relieved, as we have observed using *elt‑2* RNAi. In line with our hypothesis, RNAi of *atfs‑1*, both in the *kgb‑1*(+) control strain and in the *kgb‑1(um3)* mutant, still blocked the paraquat-triggered *hsp‑6::gfp* induction (Fig.10A, B).

From these data we conclude that we identified two functionally different groups of genes: (1) those, like *atfs‑1*, that are involved in signaling from the mitochondria to the nucleus resulting in *hsp‑6* induction, and (2) those that are involved in processes targeted by pathogen invasion and toxin attack, whose downregulation induces the cellular surveillance system and results in cSADDs, including behavioral, immune, and detoxification responses (Fig. 10C).

## DISCUSSION

### Mild stress induced by paraquat evoked the UPR^mt^ in a ROS dependent manner

In this work we analyzed the response of *C. elegans* to a low, non-lethal concentration (0.5 mM) of the ROS generator paraquat by inducing the UPR^mt^, visualized by expression of the *hsp‑6::gfp* reporter gene. Whereas higher concentration of paraquat resulted in a dramatic impact on the development of *C. elegans*, including larval arrest or rapid death, the low concentration we used in our experiments only produced a slight delay of larval development, and even extended lifespan of the animals when applied at adult stage [40]. We show here that the established ROS scavenger NAC substantially reduced *hsp‑6::gfp* induction in our protocol, suggesting that ROS generated by paraquat constitutes a toxic activity that provokes the UPR^mt^. A consequence of this toxic activity is morphological alterations in the mitochondrial architecture. Downregulation of *atfs-1* severely affected the viability of animals maintained at 0.4 mM paraquat starting at L1, suggesting that at these conditions the activation of UPR^mt^ is beneficial for *C. elegans*. We suggest that ROS induces the UPR^mt^ independently of *haf‑1*, which was been proposed to be an essential component of the UPR^mt^ after induction by ethidium bromide, *zc32* and *clk‑1(qm30)* [29].

### Novel screen for genes required for paraquat induction of *hsp‑6* revealed preferentially cSADDs genes

We conducted a genome scaled screen employing postembryonic RNAi exposure to identify genes involved in the paraquat triggered UPR^mt^. This was the first systematic analysis of genes required for the paraquat/ROS induced UPR^mt^ [22,29], and also the first protocol that allowed screening with genes with an embryonic lethal mutant phenotype [23].

Among the 54 genes we identified was *atfs-1*, previously suggested to encode a key regulator of the UPR^mt^ and activator of *hsp‑6* and *hsp‑60* transcription [29]. None of the other 54 genes had previously been implicated in the ROS induced UPR^mt^. Most of them encode proteins involved in basic cellular functions, which include components of the protein degradation and protein folding pathways, as well as translation. Accordingly, RNAi knockdown of most of them caused a pronounced delay or arrest of larval development already in the absence of paraquat, which did not affect our screening due to the postembryonic application of RNAi, but would have prevented their identification in previously described screens.

The largest family of genes identified encodes components of the small and large ribosomal subunits, as well as several factors involved in protein translation. It has been suggested that stress of both mitochondria and the endoplasmic reticulum result in the downregulation of translation. Therefore, RNAi against the ribosomal proteins, in a simple model, may prevent general translation and, thus, result in a relief of stress. In agreement with such a model, Baker et al. [32] recently suggested that, upon protein misfolding, the activation of the kinase GCN‑2 results in an inhibition of translation initiation. Thus, upon mitochondrial stress, blocking translation would reduce the load on the protein folding machinery, and thereby alleviate stress. For a number of reasons downregulation of general translation was not observed by us: First, because downregulation of ribosomal genes did not prevent the expression of other GFP reporter genes tested in this study (Fig. 9, Fig. S5). Second, because we found that downregulation of *rpl‑36*, a representative member of this group of genes, showed an enhanced rather than reduced sensitivity to paraquat. Third, a general reduction of translation mediated by the *ife‑2(ok306)* mutant in our hands was not sufficient to phenocopy the effects of RNAi against ribosomal genes. Intriguingly, we find that, in addition to genes for the ribosomal subunits, most genes identified in our screen overlap with a list of genes found in a recently published report addressing food avoidance behavior as part of cSADDs (see Table S1) [3]. There, the existence of a systemic surveillance system of basic molecular functions was proposed, which triggers defensive molecular and behavioral consequences that allows animals to detect invading pathogens or exposure to toxins. RNAi of core cellular activities, which include translation and protein turnover, induces detoxification and innate immune defense already in the absence of a pathogen or pathogenic toxin.

A total of 36 of the 54 genes (67 %) identified in our screen have been linked to aversion behavior ([3], Table S1). In addition, of the remaining genes all but seven, including *atfs‑1*, encode proteins belonging to functional classes or protein complexes of which genes encoding other components have been identified in aversion behavior. Since the cellular surveillance system signals through an endocrine response which involves the activity of the JNK pathway, we tested whether inactivation of *kgb‑1* could release the inhibitory role of cSADDs. This we could show for *elt‑2* and *rpl‑36*, but not for *atfs‑1*. The knockdown of the latter did not trigger cSADDs, and therefore *atfs-1* is most likely specifically involved in UPR^mt^ signaling.

In summary, we suggest that the activation of the UPR^mt^ by ROS is monitored by the surveillance system. Down-regulating the activity any out of a large number of cSADDs inducing genes inhibits the activation of the paraquat triggered UPR^mt^, but does not affect heat shock response, UPR^ER^ or the SKN‑1 dependent ROS response. The repression of UPR^mt^ by cSADDs mediated pathogen attack seems at first glance counterintuitive, but might imply that particular stress responses are handled in a prioritized way in *C. elegans*. It would, for example, suggest that animals experiencing damage to basic cellular functions, e.g. by exposure to strong toxins, could block the response to mild mitochondrial stress, whereas other types of stress could be preferentially handled to save energy for the execution of acutely required repair processes. Another quite attractive hypothesis is that activation of cSADDs could block UPR^mt^ in order to actively raise ROS levels locally as part of an active defense strategy. The toxic properties of ROS are used in both plants and humans in immune responses against invaders in a process called active burst (for an overview [60-64]), but so far we were unable to show a local increase in ROS as a consequence of cSADDs activation. An intricate aspect of surveillance system is that it also monitors the mitochondria and their functional integrity, since we observed that established inducers of the UPR^mt^, most notably including paraquat, themselves can activate the cellular surveillance system and consecutively may elicit food aversion behavior. We verified that the 0.5 mM paraquat used in our screen did not itself trigger food avoidance, eliminating a direct interference between two stress responses. Concentration higher than 5‑10 mM paraquat, however, inevitably induced pronounced food avoidance in the worms as a consequence of cSADDs.

A number of genes have been identified that both induce *hsp‑6::gfp* and food avoidance behavior, when depleted by RNAi [3,22]. These genes encode proteins essential for main mitochondrial functions, such as cytochrome oxidase, ATP synthase, and HSP-6. Because cSADDs induced by these serious mitochondrial impairment induce UPR^mt^ instead of blocking it, we conclude, that they must activate the cellular surveillance system by a variant mechanism, which prevents blocking of UPR^mt^ (Fig. 10). In summary, during mild mitochondrial stress the cellular surveillance system suppresses the induction of UPR^mt^ to benefit from the remaining mitochondrial activity for other stress compensatory functions, whereas in case of severe mitochondrial stress the induction of UPR^mt^ is favored in order to maintain an essential level mitochondrial metabolism.

A recently published list suggests that genes encoding key factors of mitochondrial biogenesis, mitochondrial fission, and mitophagy are induced through the UPR^mt^ [29]. Because these are resource consuming processes, it is conceivable that the cSADDs downregulate the UPR^mt^ in case of residual mitochondrial function in order to allocate these resources to other defense mechanisms optimizing the benefit for the cell.

MATERIAL AND METHODS

### Transgenic and mutant *C. elegans* strains

*C. elegans* variety Bristol, strain N2 was used as wild type strain. All strains were maintained and raised at 20°C on NGM agar seeded with *Escherichia coli* OP50 [65], unless otherwise indicated. The following strains were obtained from CGC: SJ4100: zcIs13[*Phsp‑6::GFP*], SJ4005: zcIs4[*Phsp‑4::GFP*], CL2166: dvIs19[*pAF15(gst‑4::GFP::NLS)*], ST66: ncIs17[*Phsp‑16.2::eGFP* + pBluescript], RB867: *haf‑1(ok705)*IV, KX15: *ife‑2(ok306)*X, ZG31: *hif‑1(ia4)*V, CF1038: *daf‑16(mu86)*I, EU1: *skn‑1(zu67)*IV/nT1[*unc ?(n754)let ?*](IV;V), DP38: *unc‑119(ed3)*III, MQ887: *isp‑1(qm150)*IV. The following strain was obtained by backcrossing SJ4100 seven times against laboratory N2: BR5194: zcIs13[*Phsp‑6::GFP*]. The following strains were obtained by crossing the respective mutants (see above) with BR5194: BR6118: *haf‑1(ok705)*; zcIs13[*Phsp‑6::gfp*], BR6019: *ife‑2(ok306)*; zcIs13[*Phsp‑6::gfp*], BR6097: *hif‑1(ia4)*; zcIs13[*Phsp‑6::GFP*], BR6020: *daf‑16(mu86)*; zcIs13[*Phsp‑6::gfp*], BR6098: *skn‑1(zu67)*/nT1; zcIs13[*Phsp‑6::gfp*], BR 6372: *isp‑1(qm150)*; zcIs13[*Phsp‑6::gfp*] The reporter strain SJ4058: zcIs9[*Phsp‑60::gfp*] was obtained from C. Benedetti. The UPR^mt^ reporter strain SJ52: [*zc32* II; *hsp‑60::gfp* V] was kindly provided by C. Haynes.

### RNA interference assays

1) RNAi screening protocol: Genome-scaled screening was performed in duplicated 96 well liquid culture plates using the ORFeome RNAi feeding library (Open Biosystems) [55]. Day 1: RNAi bacteria from frozen glycerol stocks were inoculated in 40 µl LB supplemented with 12.5 µg/ml tetracycline and 12.5 µg/ml carbenicillin and grown at 28°C, 180 rpm overnight. Eggs were prepared from gravid *C. elegans* adults by alkaline sodium hypochlorite treatment and allowed to develop in M9 at 15°C overnight. Day 2: 1.0 mM IPTG was added and the incubation was continued for another 2 h at 37°C, 180 rpm. A suspension of synchronized *C. elegans* L1 larvae was diluted to a concentration of 1 worm/µl in M9 supplemented with 10 µg/ml cholesterol, 50 µg/ml carbenicillin, 12 µg/ml tetracycline, 1 mM IPTG and 10 µg/ml fungizone. 20 µl of this suspension were distributed to 96 well plates after the bacterial culture had cooled down to room temperature. Day 3: paraquat (Sigma) was added to each well to a final concentration of 2.0 mM. Day 5: Plates were screened for non-GFP-expressing worms. Positive RNAi bacteria were recloned, sequenced and retested in at least one additional liquid culture test and subsequently also on RNAi NGM agar plates (see below).

2) RNAi on NGM plates: A 1:50 dilution of the respective RNAi bacterial overnight culture (37°C, 150 rpm) in LB medium supplemented with 12.5 µg/ml tetracycline and 12.5 µg/ml carbenicillin was grown for another 6 h at 37°C, 150 rpm. Bacteria were then seeded on NGM plates containing 1.0 mM IPTG and 25.0 mg/ml carbenicillin.

### Stress induction on NGM agar plates

Eggs were prepared from the respective gravid *C. elegans* adults by exposure to alkaline sodium hypochlorite and allowed to hatch in M9 [65] overnight. Synchronized L1 larvae were placed on NGM agar plates seeded with the respective bacteria.

1) Heat shock assay: L1 larvae (ST66) were grown on the respective RNAi bacteria for two days, subjected to 34°C for 4 h and analyzed for GFP expression one day later.

2) Paraquat/Acrylamide stress assays: L1 larvae (BR5194, CL2166) were grown on the respective RNAi bacteria for 24 h, subjected to 0.5 mM paraquat (Sigma), 0.25 µM rotenone (Sigma), 0.25 µM antimycin A (Sigma), or 2.1 mM acrylamide (BioRad), respectively. Chemicals were added from aqueous stock solutions (for rotenone in 0.1% dimethylsulfoxide (DMSO)) onto the plates. An influence of DMSO on the investigated stress signaling has been ruled out. GFP expression was analyzed two days later.

3) UPR^ER^ assay: L1 worms (SJ4005) were immediately exposed to 7.2 µM tunicamycin (Sigma) after being placed on the respective RNAi bacteria and analyzed for GFP expression three days later.

4) *zc32* stress assay: L1 larvae (SJ52) were grown on the respective RNAi bacteria at 15°C, subjected to the restrictive temperature of 25°C as soon as animals raised on control L4440 plates had developed to L4/young adults and analyzed for GFP after two days.

### NAC assay

Day 1: N-acetyl-L-cysteine (NAC) (Sigma) aqueous stock solution (200.0 mM) was distributed to NGM agar plates to a final concentration of 15 mM. Gravid adults were left to lay eggs on NAC plates for 6 h. Day 3: Paraquat was added to the plates (0.5 mM). Day 5: GFP fluorescence was quantified.

### Paraquat resistance test

Eggs were prepared from gravid *C. elegans* adults (N2) by exposure to alkaline sodium hypochlorite and allowed to hatch in M9 [65] over night. Synchronized L1 larvae were placed on NGM agar plates seeded with the respective bacteria and containing 0.4 mM paraquat. Worms were raised at 20°C for five days. Then the number of animals that reached the adult stage and the number of animals which still remained in larval stages were determined.

### Staining of mitochondria

Lyophilized Mito Tracker stain (Mitotracker Deep Red FM, Invitrogen) was suspended in anhydrous dimethylsulfoxide to a stock solution of 1 M, which was diluted further in H_2_0 to a working solution of 10 mM. Working solution was added to the worms on NGM agar plates to a final concentration of 100 nM 8 h prior to analyses.

### Microscopy and image analysis

Live worms were analyzed for GFP expression either on NGM agar plates or in 96 well microtiter plates in liquid with a stereo microscope (SZX12, Olympus). Micrographs were taken from cold-immobilized animals on NGM plates using the stereo microscope and a Zeiss MRm2 CCD camera. For quantification micrographs were taken from sodium azide-immobilized animals with an Axioimager.Z1 compound microscope with an AxioCam MRm3 CCD camera; Axiovision software version 4.8.1 (Carl Zeiss AG, Germany) was used for image analysis. Mito Tracker stained mitochondria were analyzed with a Nikon Ti A1 confocal microscope and NIS-Elements AR 4.0 64-bit software using a 60x water immersion objective with a numerical aperture of 1.2.

### Food aversion assay

RNAi: Assay plates were prepared as described [3]. Synchronized L1 staged N2 were placed in the middle of the bacterial lawn. Aversion was analyzed after 48 h and scored by the quotient of the amount of animals residing outside the bacterial lawn (N_off_) and the total amount of animals (N_total_) (aversion score (AV): N_off_/N_total_). Empty vector L4440 RNAi bacteria and *elt‑2* RNAi expressing bacteria were used as controls [3].

### Statistical analysis

Statistical analyses were performed with GraphPad Prism 4 software using unpaired t test (with Welch's correction if required), one-way analysis of variance (plus Tukey's multiple comparison test), Mann-Whitney test or Kruskal-Wallis test (plus Dunn's multiple comparison test), respectively. For the comparison of data sets with more than one parameter (RNAi and drug treatment) the background expression of the non-drug treated RNAi-fed cohort was subtracted from the respective drug-treated cohort prior to analyses if not stated otherwise.

## ACKNOWLEDGMENTS

We thank Cole Haynes for generously providing the UPR^mt^ reporter strain (SJ52) and advice in its handling. We thank Daniel Garzke for assistance during RNAi screening, Ruth Jähne and Simone Werner for assistance in stress assays, and Lydia Lieb for technical assistance. Special thank goes to Rebecca Dickstein and Pamela Padilla from University of North Texas at Denton, and to Justine Melo from MGH/Harvard Medical School for helpful discussions.

## REFERENCES

1. Przybysz AJ, Choe KP, Roberts LJ, Strange K (2009) Increased age reduces DAF-16 and SKN-1 signaling and the hormetic response of Caenorhabditis elegans to the xenobiotic juglone. Mech Ageing Dev 130: 357-369.

2. Bellier A, Chen CS, Kao CY, Cinar HN, Aroian RV (2009) Hypoxia and the hypoxic response pathway protect against pore-forming toxins in C. elegans. PLoS Pathog 5: e1000689.

3. Melo JA, Ruvkun G (2012) Inactivation of Conserved C. elegans Genes Engages Pathogen- and Xenobiotic-Associated Defenses. Cell 149: 452-466.

4. Partridge FA, Tearle AW, Gravato-Nobre MJ, Schafer WR, Hodgkin J (2008) The C. elegans glycosyltransferase BUS-8 has two distinct and essential roles in epidermal morphogenesis. Dev Biol 317: 549-559.

5. Lindquist S, Craig EA (1988) The heat-shock proteins. Annu Rev Genet 22: 631-677.

6. Patil C, Walter P (2001) Intracellular signaling from the endoplasmic reticulum to the nucleus: the unfolded protein response in yeast and mammals. Curr Opin Cell Biol 13: 349-355.

7. Walter P, Ron D (2011) The unfolded protein response: from stress pathway to homeostatic regulation. Science 334: 1081-1086.

8. Sorger PK (1991) Heat shock factor and the heat shock response. Cell 65: 363-366.

9. Link CD, Cypser JR, Johnson CJ, Johnson TE (1999) Direct observation of stress response in Caenorhabditis elegans using a reporter transgene. Cell Stress Chaperones 4: 235-242.

10. Link CD, Johnson CJ (2002) Reporter transgenes for study of oxidant stress in Caenorhabditis elegans. Redox Cell Biology and Genetics, Pt B 353: 497-505.

11. Snutch TP, Baillie DL (1983) Alterations in the pattern of gene expression following heat shock in the nematode Caenorhabditis elegans. Can J Biochem Cell Biol 61: 480-487.

12. Takatsuki A, Arima K, Tamura G (1971) Tunicamycin, a new antibiotic. I. Isolation and characterization of tunicamycin. J Antibiot (Tokyo) 24: 215-223.

13. Shen X, Ellis RE, Lee K, Liu CY, Yang K, et al. (2001) Complementary signaling pathways regulate the unfolded protein response and are required for C. elegans development. Cell 107: 893-903.

14. Tullet JM, Hertweck M, An JH, Baker J, Hwang JY, et al. (2008) Direct inhibition of the longevity-promoting factor SKN-1 by insulin-like signaling in C. elegans. Cell 132: 1025-1038.

15. Oh SW, Mukhopadhyay A, Dixit BL, Raha T, Green MR, et al. (2006) Identification of direct DAF-16 targets controlling longevity, metabolism and diapause by chromatin immunoprecipitation. Nat Genet 38: 251-257.

16. Dong MQ, Venable JD, Au N, Xu T, Park SK, et al. (2007) Quantitative mass spectrometry identifies insulin signaling targets in C. elegans. Science 317: 660-663.

17. Schuster E, McElwee JJ, Tullet JM, Doonan R, Matthijssens F, et al. (2010) DamID in C. elegans reveals longevity-associated targets of DAF-16/FoxO. Mol Syst Biol 6: 399.

18. Oliveira RP, Porter Abate J, Dilks K, Landis J, Ashraf J, et al. (2009) Condition-adapted stress and longevity gene regulation by Caenorhabditis elegans SKN-1/Nrf. Aging Cell 8: 524-541.

19. Okuyama T, Inoue H, Ookuma S, Satoh T, Kano K, et al. (2010) The ERK-MAPK pathway regulates longevity through SKN-1 and insulin-like signaling in Caenorhabditis elegans. J Biol Chem 285: 30274-30281.

20. Wang J, Robida-Stubbs S, Tullet JM, Rual JF, Vidal M, et al. (2010) RNAi screening implicates a SKN-1-dependent transcriptional response in stress resistance and longevity deriving from translation inhibition. PLoS Genet 6.

21. Sykiotis GP, Habeos IG, Samuelson AV, Bohmann D (2011) The role of the antioxidant and longevity-promoting Nrf2 pathway in metabolic regulation. Curr Opin Clin Nutr Metab Care 14: 41-48.

22. Yoneda T, Benedetti C, Urano F, Clark SG, Harding HP, et al. (2004) Compartment-specific perturbation of protein handling activates genes encoding mitochondrial chaperones. Journal of Cell Science 117: 4055-4066.

23. Benedetti C, Haynes CM, Yang Y, Harding HP, Ron D (2006) Ubiquitin-like protein 5 positively regulates chaperone gene expression in the mitochondrial unfolded protein response. Genetics 174: 229-239.

24. Haynes CM, Petrova K, Benedetti C, Yang Y, Ron D (2007) ClpP mediates activation of a mitochondrial unfolded protein response in C. elegans. Dev Cell 13: 467-480.

25. Haynes CM, Yang Y, Blais SP, Neubert TA, Ron D (2010) The matrix peptide exporter HAF-1 signals a mitochondrial UPR by activating the transcription factor ZC376.7 in C. elegans. Mol Cell 37: 529-540.

26. Pellegrino MW, Nargund AM, Haynes CM (2012) Signaling the mitochondrial unfolded protein response. Biochim Biophys Acta.

27. Haynes CM, Ron D (2010) The mitochondrial UPR - protecting organelle protein homeostasis. J Cell Sci 123: 3849-3855.

28. Durieux J, Wolff S, Dillin A (2011) The cell-non-autonomous nature of electron transport chain-mediated longevity. Cell 144: 79-91.

29. Nargund AM, Pellegrino MW, Fiorese CJ, Baker BM, Haynes CM (2012) Mitochondrial Import Efficiency of ATFS-1 Regulates Mitochondrial UPR Activation. Science.

30. Hartl FU, Martin J, Neupert W (1992) Protein folding in the cell: the role of molecular chaperones Hsp70 and Hsp60. Annu Rev Biophys Biomol Struct 21: 293-322.

31. Broadley SA, Hartl FU (2008) Mitochondrial stress signaling: a pathway unfolds. Trends Cell Biol 18: 1-4.

32. Baker BM, Nargund AM, Sun T, Haynes CM (2012) Protective Coupling of Mitochondrial Function and Protein Synthesis via the eIF2alpha Kinase GCN-2. PLoS Genet 8: e1002760.

33. Nistico R, Mehdawy B, Piccirilli S, Mercuri N (2011) Paraquat- and rotenone-induced models of Parkinson's disease. Int J Immunopathol Pharmacol 24: 313-322.

34. Yang W, Hekimi S (2010) A mitochondrial superoxide signal triggers increased longevity in Caenorhabditis elegans. PLoS Biol 8: e1000556.

35. Cocheme HM, Murphy MP (2008) Complex I is the major site of mitochondrial superoxide production by paraquat. J Biol Chem 283: 1786-1798.

36. Degli Esposti M (1998) Inhibitors of NADH-ubiquinone reductase: an overview. Biochim Biophys Acta 1364: 222-235.

37. Honda Y, Honda S (1999) The daf-2 gene network for longevity regulates oxidative stress resistance and Mn-superoxide dismutase gene expression in Caenorhabditis elegans. FASEB J 13: 1385-1393.

38. Nystrom T (2005) Role of oxidative carbonylation in protein quality control and senescence. EMBO J 24: 1311-1317.

39. Feng J, Bussiere F, Hekimi S (2001) Mitochondrial electron transport is a key determinant of life span in Caenorhabditis elegans. Dev Cell 1: 633-644.

40. Lee SJ, Hwang AB, Kenyon C (2010) Inhibition of respiration extends C. elegans life span via reactive oxygen species that increase HIF-1 activity. Curr Biol 20: 2131-2136.

41. Schulz TJ, Zarse K, Voigt A, Urban N, Birringer M, et al. (2007) Glucose restriction extends Caenorhabditis elegans life span by inducing mitochondrial respiration and increasing oxidative stress. Cell Metab 6: 280-293.

42. Drechsel DA, Patel M (2009) Differential contribution of the mitochondrial respiratory chain complexes to reactive oxygen species production by redox cycling agents implicated in parkinsonism. Toxicol Sci 112: 427-434.

43. Fukushima T, Tanaka K, Lim H, Moriyama M (2002) Mechanism of cytotoxicity of paraquat. Environ Health Prev Med 7: 89-94.

44. Fukushima T, Yamada K, Isobe A, Shiwaku K, Yamane Y (1993) Mechanism of cytotoxicity of paraquat. I. NADH oxidation and paraquat radical formation via complex I. Exp Toxicol Pathol 45: 345-349.

45. Shimada H, Hirai K, Simamura E, Hatta T, Iwakiri H, et al. (2009) Paraquat toxicity induced by voltage-dependent anion channel 1 acts as an NADH-dependent oxidoreductase. J Biol Chem 284: 28642-28649.

46. Yang W, Li J, Hekimi S (2007) A Measurable increase in oxidative damage due to reduction in superoxide detoxification fails to shorten the life span of long-lived mitochondrial mutants of Caenorhabditis elegans. Genetics 177: 2063-2074.

47. Link CD, Johnson CJ (2002) Reporter transgenes for study of oxidant stress in Caenorhabditis elegans. Methods Enzymol 353: 497-505.

48. Kahn NW, Rea SL, Moyle S, Kell A, Johnson TE (2008) Proteasomal dysfunction activates the transcription factor SKN-1 and produces a selective oxidative-stress response in Caenorhabditis elegans. Biochem J 409: 205-213.

49. Calleman CJ, Bergmark E, Costa LG (1990) Acrylamide is metabolized to glycidamide in the rat: evidence from hemoglobin adduct formation. Chem Res Toxicol 3: 406-412.

50. Benrahmoune M, Therond P, Abedinzadeh Z (2000) The reaction of superoxide radical with N-acetylcysteine. Free Radic Biol Med 29: 775-782.

51. Aruoma OI, Halliwell B, Hoey BM, Butler J (1989) The antioxidant action of N-acetylcysteine: its reaction with hydrogen peroxide, hydroxyl radical, superoxide, and hypochlorous acid. Free Radic Biol Med 6: 593-597.

52. Tawe WN, Eschbach ML, Walter RD, Henkle-Duhrsen K (1998) Identification of stress-responsive genes in Caenorhabditis elegans using RT-PCR differential display. Nucleic Acids Res 26: 1621-1627.

53. An JH, Blackwell TK (2003) SKN-1 links C. elegans mesendodermal specification to a conserved oxidative stress response. Genes Dev 17: 1882-1893.

54. Lehtinen MK, Yuan Z, Boag PR, Yang Y, Villen J, et al. (2006) A conserved MST-FOXO signaling pathway mediates oxidative-stress responses and extends life span. Cell 125: 987-1001.

55. Rual JF, Ceron J, Koreth J, Hao T, Nicot AS, et al. (2004) Toward improving Caenorhabditis elegans phenome mapping with an ORFeome-based RNAi library. Genome Res 14: 2162-2168.

56. Huang da W, Sherman BT, Lempicki RA (2009) Systematic and integrative analysis of large gene lists using DAVID bioinformatics resources. Nat Protoc 4: 44-57.

57. Hawkins MG, McGhee JD (1995) elt-2, a second GATA factor from the nematode Caenorhabditis elegans. J Biol Chem 270: 14666-14671.

58. Sommermann EM, Strohmaier KR, Maduro MF, Rothman JH (2010) Endoderm development in Caenorhabditis elegans: the synergistic action of ELT-2 and -7 mediates the specification-->differentiation transition. Dev Biol 347: 154-166.

59. Hasegawa K, Miwa S, Isomura K, Tsutsumiuchi K, Taniguchi H, et al. (2008) Acrylamide-responsive genes in the nematode Caenorhabditis elegans. Toxicol Sci 101: 215-225.

60. O'Brien JA, Daudi A, Butt VS, Paul Bolwell G (2012) Reactive oxygen species and their role in plant defence and cell wall metabolism. Planta.

61. Perez-Perez ME, Lemaire SD, Crespo JL (2012) ROS and autophagy in plants and algae. Plant Physiol.

62. Suzuki N, Miller G, Morales J, Shulaev V, Torres MA, et al. (2011) Respiratory burst oxidases: the engines of ROS signaling. Curr Opin Plant Biol 14: 691-699.

63. Kumar A, Farhana A, Guidry L, Saini V, Hondalus M, et al. (2011) Redox homeostasis in mycobacteria: the key to tuberculosis control? Expert Rev Mol Med 13: e39.

64. Puertollano MA, Puertollano E, de Cienfuegos GA, de Pablo MA (2011) Dietary antioxidants: immunity and host defense. Curr Top Med Chem 11: 1752-1766.

65. Brenner S (1974) The genetics of Caenorhabditis elegans. Genetics 77: 71-94.

66. Syntichaki P, Troulinaki K, Tavernarakis N (2007) eIF4E function in somatic cells modulates ageing in Caenorhabditis elegans. Nature 445: 922-926.

67. Tavernarakis N (2007) Protein synthesis and aging: eIF4E and the soma vs. germline distinction. Cell Cycle 6: 1168-1171.

## FIGURE LEGENDS

### Figure 1: Paraquat induces *hsp‑6* and its reporter in a ROS dependent manner

A. Quantitative analysis (by qRT-PCR) of endogenous *hsp‑6* mRNA in wild type (N2) worms exposed to 0.5 mM paraquat from early L3 stage on for 4 h, 24 h and 30 h, presented as fold induction. Dots indicate single experiments; mean plus SD.

B. Quantification of GFP fluorescence intensity in the *hsp‑6* reporter strain (*Phsp‑6::gfp*) after two days of exposure to 0.5 mM paraquat from early L3 stage on. Paraquat significantly increased (p<0.0001) *hsp‑6* reporter expression. Columns represent pooled normalized values of three independent experiments plus standard error of the mean (SEM). Numbers in or on columns indicate the number of analyzed animals (n_total_=152). ****:*p<0.0001; Mann Whitney test.

C. The paraquat-triggered induction of the *hsp‑6* reporter (*Phsp‑6::gfp*) was decreased by the addition of the ROS scavenger N-acetyl-L-cysteine (NAC). Columns represent normalized values plus standard error of the mean (SEM). Numbers in columns indicate the number of analyzed animals (n_total_=40). ***: p<0.0001; Unpaired t test with Welch's correction.

### Figure 2: The induction of *hsp‑6::gfp* by paraquat does not require HAF‑1, but ATFS‑1.

A. GFP staining after induction of the *hsp‑6* reporter gene (*Phsp‑6::gfp*) with (PQ) or without (no PQ) 0.5 mM paraquat. L4440: vector control. *atfs-1(i)*: RNAi against *atfs-1*; *haf-1*(+): wild type and *haf-1*(-): *ok705* mutant allele. Equal optical settings per row. Scale bar 200 µm.

B. Quantification of GFP fluorescence intensity confirms that the *ok705* mutation did not block, but increased the induction of *hsp‑6::gfp* with paraquat, whereas knockdown of *atfs****‑****1* (*atfs****‑****1(i)*) reduced reporter fluorescence to background. Columns represent pooled normalized values of three independent experiments plus standard error of the mean (SEM). Numbers in or on columns indicate the number of analyzed animals (*ok705*: n_total_=318; *atfs****‑****1 (i)*: n_total_=339). ***: p<0.0001; Mann Whitney test (after subtraction of respective background fluorescence).

### Figure 3: Mitochondrial ROS generators induce *hsp‑6::gfp*

A. The *hsp‑6* reporter strain (*Phsp‑6::gfp*) was induced when exposed during larval development to paraquat (0.5 mM), antimycin A (0.25 µM), or rotenone (0.25 µM). A ROS generating point mutation *(qm150)* in the ETC complex III gene *isp‑1* also induced the *hsp‑6* reporter. Scale bar: 200 µm.

B. Acrylamide, whose oxidative stress generating activity has not been linked to mitochondrial metabolism, induced the *hsp‑6* reporter weakly, but paraquat induction was considerably stronger. Columns represent pooled normalized values of three independent experiments plus standard error of the mean (SEM). Numbers in or on columns indicate the number of analyzed animals (n_total_= 234). ***: p<0.0001; Kruskal-Wallis test plus Dunn's Multiple Comparison Test.

### Figure 4: Paraquat affects mitochondrial morphology, but does not provoke the unfolded protein responses of the endoplasmatic reticulum (ER) or the cytosol.

A. Representative confocal micrographs of cells in wild type worms after Mitotracker staining. Worms were exposed to 0.5 mM paraquat starting from early L3 stage. Hypodermal mitochondrial staining was analyzed after two days. Arrows indicates the location of vulva. Scale bar: 10 µm.

B-C. Quantification of GFP fluorescence intensities in a cytosolic UPR reporter strain (*Phsp‑16.2::gfp*) (B) and an UPR^ER^ reporter strain (*Phsp‑4::gfp*) after two days of exposure to 0.5 mM paraquat or to their respective inductor (heat shock: 4 h at 34°C at L4, analyzed after one day; tunicamycin: 7.2 µM at L1, analyzed after three days). Both UPR reporters were not induced by paraquat, suggesting that the compound does not affect protein folding environment in cytosol or ER, respectively. Columns represent normalized pooled values of three independent experiments plus standard error of the mean (SEM). Numbers in or on columns indicate the number of analyzed animals (cytosolic UPR n_total_=159; UPR^ER^ n_total_=188). ***: p<0.001, n.s: p>0.05; Kruskal-Wallis test plus Dunn's Multiple Comparison Test.

### Figure 5: The *hsp‑6* response to paraquat does not require the stress inducible transcription factors SKN‑1, DAF‑16 or HIF‑1.

A-C. Representative micrographs and the corresponding quantifications of the induction of *hsp‑6* reporter (*Phsp‑6::gfp*) with paraquat in *daf‑16(mu68)* (A), *skn‑1(zu67)* (B), and *hif‑1(ia4)* (C) strains. Early L3 larvae of the respective mutants carrying the *hsp‑6* reporter (*Phsp‑6::gfp*) were exposed to 0.5 mM paraquat on OP50 plates and analyzed for GFP fluorescence after two days. Equal optical settings per row. Columns represent normalized pooled values of three independent experiments each plus standard error of the mean (SEM). Numbers in or on columns indicate the number of analyzed animals (*daf‑16*: n_total_= 293, *skn‑1*: n_total_= 272, *hif‑1*: n_total=_ 278). ***: p<0.0001, *: p=0.0214, n.s.: p<0.05; Mann Whitney test (A, B), unpaired t test with Welch's correction (C) after subtraction of respective background expression. Scale bar 250 µm.

### Figure 6. Activities of *rpl‑36* and *atfs‑1* are required for the *hsp‑6* response to paraquat.

Representative micrographs (A) and quantification of GFP fluorescence intensity (B) of two screening positives (*rpl‑36*, *atfs‑1*) show a block of the paraquat triggered induction of the *hsp‑6* reporter (*Phsp‑6::gfp)* after their RNAi. Worms were raised on respective RNAi plates from L1 larval stage and exposed to 0.5 mM paraquat at early L3 stage. GFP fluorescence was analyzed after two days. Columns represent pooled normalized values of three independent experiments plus standard error of the mean (SEM). Numbers in or on columns indicate the number of analyzed animals (n_total_=485). ****:*p<0.001; Kruskal-Wallis test plus Dunn's Multiple Comparison Test; Mann Whitney test. Equal optical settings, scale bar 200 µm. *(i)*: RNAi; L4440: empty vector control.

### Figure 7: Knockdown of *rpl‑36* and *atfs‑1* suppresses the *isp‑1(qm150)* mediated induction of the *hsp‑6* reporter.

The *isp‑1(qm150)* mutant has increased levels of mitochondrial superoxide [17] and constitutively activated the *Phsp‑6* reporter (*Phsp‑6::gfp*). RNAi of *rpl-36* and *atfs-1* suppressed (p<0.001) the constitutive *hsp‑6* reporter gene induction. Representative micrographs (A) and quantification of GFP fluorescence intensity (B). *hsp‑6* reporter worms carrying the *qm150* allele were analyzed for GFP expression after one week on the respective RNAi plates. Columns represent pooled values of three independent experiments plus standard error of the mean (SEM). Numbers in columns indicate the number of analyzed animals (n_total_=244). ****:*p<0.001; Kruskal-Wallis test plus Dunn's Multiple Comparison Test. Equal optical settings per row, scale bar 100 µm. *(i)*: RNAi; L4440: empty vector control.

### Figure 8: The downregulation of *rpl‑36* and *atfs‑1* increases paraquat sensitivity

L1 staged N2 worms were placed on the respective RNAi plates containing 0.4 mM or no paraquat, development was analyzed five days later. Downregulation of both genes enhanced sensitivity towards paraquat, indicated by delayed development. Columns represent pooled values of three independent experiments in percent. Numbers on columns indicate the number of animals analyzed (n_total_=2004). *(i)*: RNAi; L4440: empty vector control.

### Figure 9: The knockdown of *rpl‑36* and *atfs‑1* does not prevent non-mitochondrial stress responses.

Worms were grown from L1 larval stage on the respective RNAi plates before being exposed to the respective stress and analyzed four days after L1.

A. A reporter strain for the SKN-1 dependent phase II response *(Pgst‑4::gfp)* was exposed to 2.1 mM acrylamide starting at early L3 stage. RNAi of *rpl-36* and *atfs-1* did not prevent reporter gene induction as compared to vector control. Columns represent pooled normalized values of four independent experiments plus standard error of the mean (SEM). Numbers in columns indicate the number of analyzed animals (n_total_=613). ***: p<0.001; Kruskal-Wallis test plus Dunn's Multiple Comparison Test; Mann Whitney test (comparison of vector with and without acrylamide). Equal optical settings, scale bar 200 µm.

B. Cytosolic UPR (heat shock) reporter worms *(Phsp‑16.2::gfp)* were exposed to 34°C for 4h at L4. The knockdown of *rpl‑36* and *atfs‑1* significantly decreased heat stress induced reporter expression (p<0.001). Columns represent pooled normalized values of two independent experiments plus standard error of the mean (SEM). Numbers in or on columns indicate the number of analyzed animals (n_total_=266). ****:*p<0.001; Kruskal-Wallis test plus Dunn's Multiple Comparison Test; Mann Whitney test (comparison of vector with and without heat shock). Equal optical settings, scale bar 100 µm.

C. The UPR^ER^ reporter strain (*Phsp‑4::gfp*) was raised from L1 stage RNAi plates (with 7.2 µg/ml tunicamycin). UPR^ER^ induction was not blocked by any RNAi tested here, but *rpl‑36* (RNAi) strongly impaired its induction (p<0.001). Columns represent pooled normalized values of four independent experiments plus standard error of the mean (SEM). Numbers in or on columns indicate the number of analyzed animals (n_total_=363). ****:*p<0.001; Kruskal-Wallis test plus Dunn's Multiple Comparison Test; Mann Whitney test (comparison of vector with and without tunicamycin). Equal optical settings, scale bar 100 µm.

*(i)*: RNAi; L4440: empty vector control.

### Figure 10: cSADDs inhibits paraquat mediated signaling to *hsp-6* through KGB-1

A-B. In *kgb‑1(um3)* mutant animals, which are cSADDs deficient, paraquat induced *hsp-6* induction is not blocked by *elt‑2* RNAi. Thus, ROS induced UPR^mt^ is activated in the absence of functional cSADDs. In contrast, *kgb‑1(um3)* does not prevent inhibition of *hsp‑6* induction by *atfs‑1* knockdown, suggesting that it functions downstream of *kgb-1* and the cSADDs. Columns represent normalized values plus standard error of the mean (SEM). Numbers in or on columns indicate the number of analyzed animals (n_total_=181). ***: p<0.001, *: p<0.05; Kruskal-Wallis test plus Dunn's Multiple Comparison Test (A). Equal optical settings, scale bar 400 µm. *(i)*: RNAi; L4440: empty vector control; +: wild type allele (B).

C. Model: Genes activating the cSADDs (cellular surveillance system) inhibit the paraquat-triggered induction of the UPR^mt^.

## SUPPLEMENTAL FIGURE AND TABLE LEGENDS

### Figure S1: Dose-response curve of paraquat and *Phsp‑6::gfp*

*Phsp‑6::gfp* reporter worms were exposed to different concentrations of paraquat (0-50 mM) for two days starting from early L3. 50 µM was lethal. GFP fluorescence intensity was analyzed with compound microscopy. A. Representative micrographs. B. Corresponding quantification. Columns represent pooled values of three independent experiments plus standard error of the mean (SEM). Numbers in columns indicate the number of analyzed animals (n_total_=725). ***: p<0.001; Kruskal-Wallis test plus Dunn's Multiple Comparison Test.

### Figure S2: *Phsp‑6::gfp* responds more sensitively to paraquat than *Phsp‑60::gfp*

Quantification of GFP fluorescence intensity in the *hsp‑6* reporter strain (*Phsp‑6::gfp*) and the *hsp‑60* reporter strain (*Phsp‑60::gfp*) after two days of exposure to 0.5 mM and 2.0 mM paraquat, respectively. Exposure started at the early L3 stage. 0.5 mM Paraquat significantly increases (p<0.0001) *hsp‑6* reporter expression, but not *Phsp‑60::gfp,* 2.0 mM paraquat induces both reporters. Columns represent mean plus standard error of the mean (SEM). Numbers in or on columns indicate the number of analyzed animals (*hsp‑6*: n_total_=30; *hsp‑6*: n_total_=29). ***: p<0.001; Kruskal-Wallis test plus Dunn's Multiple Comparison Test.

### Figure S3: Design of the genome-scaled RNAi screen

Microtiter plates including RNAi bacterial strains were grown overnight in duplicates. Each well contained RNAi bacteria specific for one *C. elegans* gene. The same day, eggs were prepared by bleaching gravid adults and allowed to further develop overnight in supplemented M9. The next day (Day 2), bacterial cultures were induced with IPTG. Subsequently, synchronized L1 larvae were added to the bacterial cultures and maintained at 20°C. At Day 3, paraquat was added. After two days (Day 5), plates were screened for worms that failed to increase GFP expression with a stereo fluorescence microscope.

### Figure S4: Knockdown of translation associated genes abolishes *hsp‑6* induction without blocking translation

The largest group of screening positives corresponds to genes encoding ribosomal proteins or other factors implicated in protein translation. In the most trivial scenario RNAi against translation associated genes reduces translation and thus prevents GFP expression from the *hsp‑6* reporter (A). This idea is contradicted by experiments in which the induction of other GFP reporters (*hsp‑16.2::gfp*, *gst‑4::gfp*, *hsp‑4::gfp*) is still possible, when translation associated genes were knocked down (Fig. 9). *rpl‑36* RNAi even slightly hyper-activated acrylamide induced expression of *gst‑4::gfp* (Fig. 9A). It is therefore unlikely that general translation is largely hampered by downregulation of ribosomal genes in our experiments.

To further affirm this conclusion we took advantage of the *ife‑2(ok306*) deletion [66,67] mutant, in which somatic translation is reduced. This mutant should mimic the effect of translation associated RNAis if the observed inhibitory effect is mitigated through a reduction of protein translation. Paraquat induced *hsp‑6* expression was not reduced, but rather increased in *ok306* mutant animals, indicating that a moderate inhibition of translation is not sufficient to prevent *hsp‑6* induction by paraquat. The hyper-induction of *hsp‑6::gfp* could be explained by an aggravation of stress caused by reduced translation of chaperones.

Taken together, we consider it unlikely that RNAi against ribosomal genes substantially reduces translation and thereby prevents *hsp-6* reporter expression. The results rather indicate a selective inhibitory response to *hsp‑6::gfp* induction.

A. Representative micrographs of *Phsp‑6* reporter (*Phsp‑6::gfp*) worms carrying the *ife‑2(ok306)* allele induced with paraquat. *ok306* causes a moderate reduction of general protein translation without causing an imbalance of ribosomal proteins [68,69]. The *hsp‑6* induction was not reduced in an *ok306* allele. Equal optical settings, scale bar 200 µm.

B. Quantification of GFP fluorescence intensity in *Phsp‑6* reporter (*Phsp‑6::gfp*) worms carrying the *ife‑2(ok306)* allele. Columns represent pooled values of three independent experiments plus standard error of the mean (SEM). Numbers in columns indicate the number of analyzed animals (n_total_=341). ****:*p<0.0001; Mann Whitney test.

*(i)*: RNAi; vector: L4440 empty vector control.

C. *ife‑2(ok306)* which does not prevent UPR^mt^ induction by paraquat also does not trigger food aversion. n = number of analyzed animals. Aversion is determined by the ratio of worms outside the bacterial lawn (N_off_) and the total amount 48–58 h of growth on RNAi bacteria (N_total_). AV score: N_off_/N_total_.

### Figure S5: The effects of RNAi of *rpl‑36* and *atfs‑1* on *zc32* mediated activation of *Phsp-60::gfp*.

Representative micrographs (A) and quantification of GFP fluorescence intensity (B). The UPR^mt^ reporter strain (*zc32; Phsp‑60::gfp*) induces the UPR^mt^ upon shift to the restrictive temperature (25°C). Being raised on the respective RNAi plates from L1, worms were shifted from the permissive temperature (15°C) to 25°C as soon as the animals grown on control RNAi plates (L4440) had developed to L4/young adults. GFP fluorescence was analyzed after two days. As expected, knockdown of *atfs-1* reduced GFP expression. This indicates the requirement of *atfs-1* for the UPR^mt^ (p<0.001). The induction of the UPR^mt^ was not reduced but rather enhanced by *rpl‑36* RNAi (p<0.001). Columns represent pooled normalized values of four independent experiments plus standard error of the mean (SEM). Numbers in or on columns indicate the number of analyzed animals (n_total_=520). ***: p<0.001; Kruskal-Wallis test plus Dunn's Multiple Comparison Test; Mann Whitney test (comparison of vector at 15°C and 25°C). Equal optical settings, scale bar 200 µm. *(i)*: RNAi; L4440: empty vector control.

### Figure S6: The cSADDs inhibit paraquat mediated signaling to *hsp-6*

The loss of the *hsp‑6::gfp* induction in *rpl‑36(RNAi)* is suppressed by mutant *kgb-1(um3)*, indicating a KGB-1 mediated repression of UPR^mt^ by cSADDs. *rpl‑36(RNAi)* was shown to be sufficient to induce cSADDs [3]. Columns represent normalized values plus standard error of the mean (SEM). ***: p<0.001; Kruskal-Wallis test plus Dunn's Multiple Comparison Test. *(i)*: RNAi; L4440: empty vector control; (+): wild type allele.

### Table S1: Knockdown of 36 of 54 screening positives were shown by Melo and Ruvkun, 2012 [3] to evoke aversion behavior.

This table displays the subset of our screening positives which were recently shown to trigger aversion [3].

## TABLES

### Table 1: 54 screening positives and their involvement in other cellular stress pathways.

| **Gene** | **Brief description** | | **UPR^mt^**  **(paraquat)** | | **phase II**  **response** | | | **UPR^cyt^** | | **UPR^ER^** | | **UPR^mt^**  **(*zc32*)** |
| --- | --- | --- | --- | --- | --- | --- | --- | --- | --- | --- | --- | --- |
| **ATP synthesis coupled proton transport** | | | | | | | | | | | | |
| *vha‑1* | vacuolar H+‑ATPase subunit | | off | | + | | | + | | + | | off |
| *vha‑2* | vacuolar H+‑ATPase subunit | | off | | + | | | + | | + | | + |
| **Cellular protein catabolic process (Proteolysis)** | | | | | | | | | | | | |
| *rpn‑7* | 19S proteasome, regulatory subunit | | off | | + | | | + | | + | | off |
| *pas‑4* | 20S proteasome, regulatory subunit | | off | | + | | | + | | + | | off |
| *pas‑7* | 20S proteasome, regulatory subunit | | off | | + | | | + | | + | | off |
| **Cellular protein metabolic process (Protein folding)** | | | | | | | | | | | | |
| *cct‑1* | cytosolic chaperonin, subunit | | off | | + | | | + | | + | | off |
| *cct‑2* | cytosolic chaperonin, subunit | | off | | + | | | + | | + | | off |
| *cct‑4* | cytosolic chaperonin, subunit | | off | | + | | | + | | + | | off |
| *cct‑5* | cytosolic chaperonin, subunit | | off | | + | | | + | | + | | off |
| **Intracellular protein transport** | | | | | | | | | | | | |
| *snap‑1* | soluble NSF attachment Protein | | off | | + | | | + | | + | | off* |
| *sec‑23* | COPII subunit | | off | | + | | | + | | + | | off |
| *apm‑1* | adaptor complexes medium subunit | | off | | + | | | + | | + | | + |
| *apb‑1* | AP‑1/AP‑2/AP‑4, beta subunit | | off | | + | | | + | | + | | off |
| *imb‑3* | nuclear transport factor | | off | | + | | | + | | + | | off* |
| *imb‑5* | nuclear transport factor | | off | | + | | | + | | + | | + |
| **mRNA splicing** | | | | | | | | | | | | |
| *snr‑1* | small nuclear ribonucleoprotein/U1snRNP | | off | | + | | | + | | + | | + |
| *snr‑2* | small nuclear ribonucleoprotein/U1snRNP | | off | | + | | | + | | + | | off |
| *snr‑6* | small nuclear ribonucleoprotein/U1snRNP | | off | | + | | | + | | + | | off |
| **Regulation of transcription, DNA‑dependent** | | | | | | | | | | | | |
| *atfs‑1* | bZip transcription factor | | off | | + | | | + | | + | | off |
| *elt‑2* | GATA‑4/5/6 transcription factor | | off | | + | | | + | | + | | off |
| **Signaling** | | | | | | | | | | | | |
| Y47D3B.1 | G‑protein coupled receptor signaling | | off | | + | | | + | | + | | + |
| **Protein translation** | | | | | | | | | | | | |
| *hel‑1* | helicase, exporting mRNA from the nucleus | | off | | + | | | + | | + | | off |
| *phi‑2* | eIF‑4A | | off | | + | | | + | | + | | + |
| *phi‑4* | mRNA splicing factor | | off | | + | | | + | | + | | off |
| *phi‑19* | polypeptide release factor 3 | | off | | + | | | + | | + | | n.d. |
| *phi‑21* | peptide chain elongation factor | | off | | + | | | + | | + | | off |
| Y65B4A.6 | predicted ATP‑dep. RNA helicase FAL1 | | off | | + | | | + | | + | | off |
| *rpl‑14* | large ribosomal subunit protein | | off | | + | | | + | | + | | + |
| *rpl‑17* | large ribosomal subunit protein | | off | | + | | | + | | + | | + |
| *rpl‑18* | large ribosomal subunit protein | | off | | + | | | + | | + | | +* |
| *rpl‑19* | large ribosomal subunit protein | | off | | + | | | + | | + | | + |
| *rpl‑22* | large ribosomal subunit protein | | off | | + | | | + | | + | | + |
| *rpl‑23* | large ribosomal subunit protein | | off | | + | | | + | | + | | + |
| *rpl‑26* | large ribosomal subunit protein | | off | | + | | | + | | + | | + |
| *rpl‑30* | large ribosomal subunit protein | | off | | + | | | + | | + | | + |
| *rpl‑31* | large ribosomal subunit protein | | off | | + | | | + | | + | | + |
| *rpl‑33* | large ribosomal subunit protein | | off | | + | | | + | | + | | + |
| *rpl‑35* | large ribosomal subunit protein | | off | | + | | | + | | + | | + |
| *rpl‑36* | large ribosomal subunit protein | | off | | + | | | + | | + | | + |
| *rpl‑41* | large ribosomal subunit protein | | off | | + | | | + | | + | | + |
| *rps‑2* | | small ribosomal subunit protein | | off | | + | + | | + | | n.d. | |
| *rps‑7* | | small ribosomal subunit protein | | off | | + | + | | + | | + | |
| *rps‑8* | | small ribosomal subunit protein | | off | | + | + | | + | | off | |
| *rps‑14* | | small ribosomal subunit protein | | off | | + | + | | + | | off | |
| *rps‑17* | | small ribosomal subunit protein | | off | | + | + | | + | | off | |
| *rps‑26* | | small ribosomal subunit protein | | off | | + | + | | + | | off | |
| *rps‑27* | | small ribosomal subunit protein | | off | | + | + | | + | | off | |
| **Others** | |  | |  | |  |  | |  | |  | |
| *act‑3* | | actin | | off | | + | + | | + | | off* | |
| C14B1.2 | |  | | off | | + | + | | + | | off* | |
| C18A3.3 | | contains eukaryotic rRNA proc. domain | | off | | + | + | | + | | + | |
| C23G10.8 | |  | | off | | + | + | | + | | + | |
| *pan‑1* | | predicted transmembrane protein | | off | | + | + | | + | | +* | |
| W04A4.5 | | contains HEAT domain | | off | | + | + | | + | | off | |
| Y39B6A.42 | |  | | off | | + | + | | + | | + | |
| control | | L4440 empty vector | | + | | + | + | | + | | + | |

Qualitative analyses of GFP induction (by stereomicroscopy) of the different stress reporters with their respective inducers. UPR^mt^ (paraquat): induction of the *hsp‑6* reporter (*Phsp‑6::gfp*) with paraquat; phase II response: SKN‑1 dependent induction of the *gst‑4* reporter (*Pgst‑4::gfp*) with acrylamide; UPR^cyt^: induction of the *hsp‑16.2* reporter (*Phsp‑16.2::gfp*) with heat shock; UPR^ER^: induction of the *hsp‑4* reporter (*Phsp‑4::gfp*) with tunicamycin; UPR^mt^ (*zc32*): induction of the *hsp‑60* reporter (*Phsp‑60::gfp*) in the *zc32* ts-mutant at the restrictive temperature. +: GFP induced; off: GFP not induced; n.d.: not determined; *: in two out of three experiments; assessed by qualitative compound microscopy. Group assignment of individual candidates is based on DAVID gene enrichment analyses [56].

### Table 2: Knockdown of ATFS‑1 does not evoke aversion behaviour [3].

| **Gene** | **Brief description** | **N_total_** | **Aversion Score (N_off_**/**N_total_)** |
| --- | --- | --- | --- |
| *atfs‑1* | bZip transcription factor | 223 | 0.01 |
| *elt‑2* | positive control | 32 | 0.72 |
| L4440 | negative control | 309 | 0.00 |

N_total_ = total number of analyzed animals

Aversion is determined by the ratio of worms outside the bacterial lawn (N_off_) and the total amount (N_total_) 48–58 hr of growth on RNAi bacteria. Aversion scores for *atfs‑1*RNAi and L4440 represent mean value from three independent experiments.
